# Short- and long-term impact of vaccination against cytomegalovirus: a modeling study

**DOI:** 10.1186/s12916-020-01629-3

**Published:** 2020-07-02

**Authors:** Ganna Rozhnova, Mirjam E. Kretzschmar, Fiona van der Klis, Debbie van Baarle, Marjolein Korndewal, Ann C. Vossen, Michiel van Boven

**Affiliations:** 1 Julius Center for Health Sciences and Primary Care, University Medical Center Utrecht, Utrecht University, Utrecht, The Netherlands; 2 Center for Infectious Disease Control, National Institute of Public Health and the Environment, Bilthoven, The Netherlands; 3 Department of Medical Microbiology, Leiden University Medical Center, Leiden, The Netherlands

**Keywords:** Cytomegalovirus, CMV, Congenital CMV infection, Transmission model, Vaccination strategies, Hygiene intervention, CMV elimination, CMV disease burden

## Abstract

**Background:**

Infection with cytomegalovirus (CMV) is highly prevalent worldwide and can cause severe disease in immunocompromised persons and congenitally infected infants. The disease burden caused by congenital CMV infection is high, especially in resource-limited countries. Vaccines are currently under development for various target groups.

**Methods:**

We evaluated the impact of vaccination strategies and hygiene intervention using transmission models. Model parameters were estimated from a cross-sectional serological population study (*n*=5179) and a retrospective birth cohort (*n*=31,484), providing information on the age- and sex-specific CMV prevalence and on the birth prevalence of congenital CMV (cCMV).

**Results:**

The analyses show that vertical transmission and infectious reactivation are the main drivers of transmission. Vaccination strategies aimed at reducing transmission from mother to child (vaccinating pregnant women or women of reproductive age) can yield substantial reductions of cCMV in 20 years (31.7–71.4% if 70% of women are effectively vaccinated). Alternatively, hygiene intervention aimed at preventing CMV infection and re-infection of women of reproductive age from young children is expected to reduce cCMV by less than 2%. The effects of large-scale vaccination on CMV prevalence can be substantial, owing to the moderate transmissibility of CMV at the population level. However, as CMV causes lifelong infection, the timescale on which reductions in CMV prevalence are expected is in the order of several decades. Elimination of CMV infection in the long run is only feasible for a vaccine with a long duration of protection and high vaccination coverage.

**Conclusions:**

Vaccination is an effective intervention to reduce the birth prevalence of cCMV. Population-level reductions in CMV prevalence can only be achieved on a long timescale. Our results stress the value of vaccinating pregnant women and women of childbearing age and provide support for the development of CMV vaccines and early planning of vaccination scenarios and rollouts.

## Background

Human cytomegalovirus (CMV) is a highly prevalent herpesvirus with lifetime probability of infection that ranges from 50 to 100% in populations throughout the world [1]. Primary infection is usually mild or even asymptomatic. However, in infants with congenital CMV infection (cCMV) and in recipients of solid organ and stem cell transplants, the risk of severe disease is high [2–5]. Moreover, CMV has been linked to low-grade inflammation, atherosclerosis, and associated diseases [6]; requires considerable resources of the host’s immune system [7, 8]; and could negatively affect the effectiveness of vaccination against other pathogens in elderly [9].

The burden of disease caused by CMV infection worldwide is high. In the USA, an estimated 7000 children per year are affected by disease caused by cCMV, which is more than for other congenital disorders [10]. In a systematic review of studies in developing countries, Lanzieri et al. [11] found that maternal seroprevalence ranged from 84 to 100% and CMV birth prevalence from 0.6 to 6.1%, which is considerably higher than rates reported for Europe and North America [12]. Estimates of disability-adjusted life years (DALYs) due to cCMV are scarce. In a study from Belgium, the burden of disease due to cCMV was estimated at 18 DALYs per 100,000 in Belgium for 2013, and ten times higher than the burden caused by congenital toxoplasmosis [13]. An estimated 25% of cases of hearing loss in young children in the USA has been attributed to cCMV [14]. The high prevalence of maternal CMV infection and incidence of cCMV worldwide [3, 15–18] has motivated the development of vaccines [19, 20]. No vaccine is as yet registered, but several candidate vaccines are well past the stage of early development and are being evaluated in clinical trials [20–22]. Another preventive option to reduce cCMV is hygiene intervention [23–26], whose effectiveness among seronegative pregnant women with exposure to young children has been supported by several studies [23, 25].

The transmission of CMV from an infected to uninfected host can occur in direct contact [27] (mediated by urine or saliva), from mother to child during pregnancy [28, 29], or via breastfeeding [3, 30, 31]. In addition, direct transmission does occur not only during primary infection of a newly infected host, but also after reactivation of the virus or re-infection in a latently infected host [32]. Hence, the transmission dynamics of CMV is complex, and there is at best a partial understanding of the quantitative contributions of each of the transmission routes to the epidemiology of CMV. Such a quantitative insight is essential for proper evaluation of the impact of interventions. We take a step in this direction, by evaluating vaccination strategies and hygiene intervention with a transmission model calibrated to two large population studies from the Netherlands [33, 34].

Modeling studies on the CMV transmission dynamics are scarce. Previous studies focused on the cost-effectiveness of CMV vaccination [35, 36] or evaluated the impact of vaccination on reducing cCMV with transmission models [37–40]. However, these models did not include all known transmission routes (e.g., reactivation and multiple re-infections [37, 40]). Furthermore, the potential impact of vaccinating pregnant women and hygiene intervention aimed at preventing CMV (re-)infection of women of reproductive age from young children [25] has not been assessed. In addition, most models were parameterized using CMV seroprevalence among the population from the USA, not stratified by sex [38–40], which in some studies was limited to persons with ages between 0 and 49 years [38, 40]. Due to uncertainties in key epidemiological parameters, predictions of these models vary widely.

Our model includes direct transmission after primary infection, re-infection, or reactivation, and vertical transmission (congenitally and postnatally, defined here as transmission in the first 6 months of life, mainly via breastfeeding). The model is an extension of an earlier developed model, allowing for multiple lifetime re-infection and reactivation events [41]. To our knowledge, this is the first model to include all known transmission routes. The model parameters are estimated using data from a birth cohort and a large population-based serological study among persons with a wide age range (0–80 years) [33, 34]. Based on quantitative estimates of key parameters, we evaluate the effectiveness of a suite of vaccination strategies and hygiene intervention decreasing infectious contacts of women of reproductive age and young children. Specifically, we consider vaccination during pregnancy and several population-wide vaccination strategies (in children, adolescents, or adults; women only vs men and women), using varying vaccination coverages and durations of protection after vaccination.

We report results on the reduction in the birth prevalence of cCMV and prevented disease burden within 20 years of the start of interventions. We also provide insight into whether or not the elimination of CMV from the population is possible for each intervention. Finally, we discuss the implications of our findings for future implementation of interventions.

## Methods

### Overview

Estimates of epidemiological parameters were obtained by fitting a transmission model to data from (1) a cross-sectional population-based serological study proving age- and sex-specific seroprevalence (*n*=5179) [33] and (2) a nation-wide retrospective birth cohort study providing the birth prevalence of cCMV (*n*=31,484), both from the Netherlands [34, 42]. The model, equipped with parameters estimated from these two studies, was subsequently used to investigate the effectiveness of different interventions.

### Data

The cross-sectional population-based serological study was carried out in the Netherlands in 2006 and 2007 [33, 43]. A total of 40 out of 467 municipalities were randomly selected with probabilities proportional to their population size. From these municipalities, an age-stratified sample was drawn from the population register, and 19,781 persons were invited to complete a questionnaire and to donate a blood sample. Serum samples and questionnaires were obtained from 6382 participants. We excluded infants younger than 6 months to avoid interference with maternal antibodies and non-western migrants to preclude confounding by ethnicity. The final sample (*n*=5179) included 2842 women and 2337 men.

In the retrospective birth cohort, children born in the Netherlands between 1 January 2008 and 1 October 2008 were eligible for entry [34, 42]. Informed consent for retrieving the dried blood spot sample taken shortly after birth was obtained from 31,484 participants. Of children tested during the study period, 154 (0.5*%*) were confirmed positive by CMV DNA PCR. The birth cohort initially had been set up to study the long-term sequelae and disease burden of cCMV [34, 42].

Our analyses made use of demographic composition and fertility rates in 2017 from Statistics Netherlands (https://www.cbs.nl) and age- and sex-specific contact data [41, 44].

### Transmission model

**Fig. 1:**
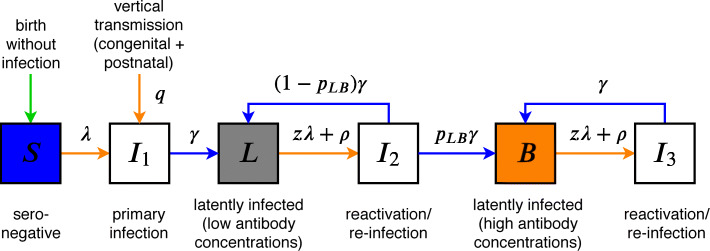
Schematic of the transmission model. Shown are epidemiological transitions in the transmission model without vaccination. Seronegative persons (susceptible, *S*) acquire primary infection (*I*_1_) either vertically from their seropositive mothers (with probability *q*) or horizontally (with the force of infection *λ*) via contact with acutely infected persons in three infectious classes (*I*_1_, *I*_2_, *I*_3_). After primary infection (duration 1/*γ* year), persons become latently infected with low antibody concentrations (latent, *L*). In this class, re-infection occurs at a rate *zλ*, where *z* is the reduction in susceptibility to re-infection in latently infected persons compared to seronegative persons, and reactivation occurs at a rate *ρ*. After reactivation/re-infection (duration 1/*γ* year), persons transit to the latent class with high antibody concentrations (boosted, *B*) with probability *p*_*LB*_, where further re-infection and reactivation events can occur (*I*_3_). Note that vertical transmission from mother to child includes both congenital and postnatal transmission via breastfeeding. The population is stratified by sex and age, and the forces of infection and reactivation rates are age- and sex-specific (not shown). Figure S1 and Figure S2 give schematics of the full model with vaccination

We developed a deterministic compartmental model describing CMV transmission in a population stratified by CMV infection status, sex, and age (Fig. 1). The model includes direct transmission from persons who experience a primary infection, a re-infection, or a reactivation episode and vertical transmission (congenital and postnatal via breastfeeding). Persons are classified as seronegative (susceptible, *S*) and seropositive who can be latently infected with low (latent, *L*) or with high antibody concentrations (boosted, *B*), or acutely infected in three infectious classes (*I*_1_ to *I*_3_) corresponding to primary infection (*I*_1_), and re-infection or reactivation from the *L* and *B* class (*I*_2_ and *I*_3_). The force of infection, *λ*, and reactivation rate, *ρ*, are age- and sex-specific (not shown). The force of infection is given by a weighted sum of the fraction of the population in the three infectious classes. Latently infected persons can be re-infected at a rate *zλ*, where *z* is the reduction in susceptibility to re-infection in latently infected persons compared to seronegative persons. Vertical transmission occurs with probability *q* from seropositive women (classes *L*, *B*, *I*_1_, *I*_2_, and *I*_3_). Congenital transmission occurs with probability *q*_*c*_ from acutely infected women (*I*_1_, *I*_2_, and *I*_3_).

### Parameter estimation

Estimation of model parameters followed a previous study [41, 45]. Here, we extended the earlier analyses by (i) allowing for multiple reactivation and re-infection events occurring over a person’s life, (ii) using cubic B-splines for flexible estimation of the age-dependent reactivation rates, and (iii) including the birth cohort data to enable estimation of the probability of cCMV. For horizontal transmission, we used an age- and sex-specific contact matrix with 17 age classes [41, 44]. The model is fitted to the data using the Hamiltonian Monte Carlo method as implemented in Stan (https://www.mc-stan.org) [46]. Details are given in Additional file 1: Appendix.

### Intervention scenarios

We considered several intervention strategies [20, 23–26, 47], namely hygiene measures [23, 25] aimed at preventing CMV (re-)infection of women of reproductive age from young children, vaccination during pregnancy reducing the probability of vertical transmission, and a suite of universal vaccination strategies [20, 47] with varying proportions of effectively vaccinated persons, ages at vaccination, sexes to be vaccinated, and durations of protection.

For universal vaccination, we further distinguished between scenarios in which the vaccine is assumed to protect only against primary infection in seronegative persons (“prevention of infection”) or against primary infection in seronegative persons and re-infection/reactivation in seropositive persons (“prevention of (re-)infection and reactivation”) [20, 47]. The target population for vaccination was either infants in the first year of life, adolescent boys and girls at the age of 10 years, adolescent girls at the age of 10 years, or women of reproductive age (15–50 years).

In the baseline scenario, we assumed that the proportion of effectively vaccinated persons (henceforth called effectively vaccinated proportion = vaccination coverage × vaccine efficacy) was 70%. The average duration of protection by the vaccine was 10 years. Hygiene measures assumed a 70% reduction in infectious contacts between 15–50-year-old women and 0–5-year-old children.

Main outcome measures were the reduction in the incidence of cCMV, primary infection and re-infection/reactivation, and DALYs prevented after 20 years. In addition, we evaluated the long-term impact of an intervention by computing the effective reproduction number and (critical) proportion of persons who must be effectively vaccinated to eliminate CMV from the population [45, 48, 49]. The effective reproduction number quantifies the intervention effort necessary for disease elimination. In theory, a disease is eliminated whenever this number is below 1 [45, 48].

The model was implemented using a system of ordinary differential equations for 16 5-year age groups and a group of 0–6-month-old infants (Additional File 1: Appendix). All interventions were introduced from the endemic equilibrium. The disease burden for the Netherlands was computed using an estimate of 3.034 (95%CrI 1.202–6.105) DALYs per case of cCMV [13].

### Sensitivity analyses

We conducted sensitivity analyses for the effectively vaccinated proportion and duration of protection after vaccination. Specifically, we varied the proportion from 0 to 100% and the duration of protection from 2.5 years to lifelong. In addition, we present parameter estimation and intervention results for a different set of prior distributions of the reactivation rates (Figure S4, Figure S5 and Table S2).

## Results

### Parameter estimation

**Fig. 2:**
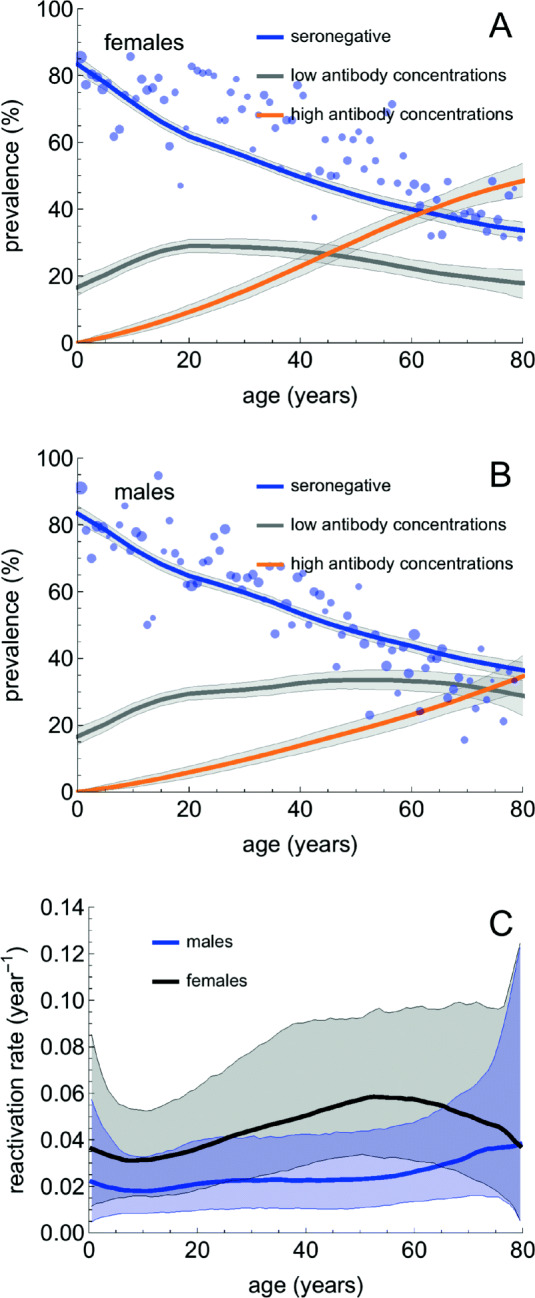
Overview of the estimation results. **a**, **b** The estimated age-specific prevalence of seronegative persons, seropositive persons with low antibody concentrations, and seropositive persons with high antibody concentrations in females (**a**) and males (**b**), respectively. **c** The age-specific reactivation rates per year for females (black) and males (blue), respectively. The solid lines represent the estimated medians, and the shaded regions correspond to 95% credible intervals obtained from 1000 parameter samples from the posterior distribution. The circles are seroprevalence data [33] indicating the fraction of samples that would be classified as seronegative with the cut-off specified by the supplier of the assay. The circle size gives a measure for the number of samples in 1-year age groups. The number of samples per 1-year age group is approximately 35 (females) and 30 (males). Note that the seroprevalence is estimated with high precision and that credible intervals for the reactivation rates are broad

Figure 2 a and b show the estimated CMV prevalence (solid lines) in females and males as a function of age. At the age of 6 months, about 17.2% (95%CrI 14.7–20.0%) of females and males were estimated to be seropositive. The prevalence of seronegative persons decreased gradually with age to 33.7% (95%CrI 31.2–36.2%) and 36.5% (95%CrI 33.9–38.8%) at 80 years in females and males, respectively. The estimated prevalence of persons with low antibody concentrations varied from 16.6 to 29.0% in females and from 16.6 to 33.6% in males. The prevalence of persons with high antibody concentrations gradually increased with age, reaching 48.4% (95%CrI 43.7–53.7%) at 80 years in females and 34.7% (95%CrI 29.7–41.0%) in males.

The estimated reactivation rates ranged from 0.031 to 0.058 per year for females and from 0.018 to 0.038 per year for males (Fig. 2c). For all ages, the reactivation rate was higher in females than in males. The estimated probabilities of vertical transmission and of cCMV from acutely infected pregnant women were 0.37 (95%CrI 0.31–0.43) and 0.17 (95%CrI 0.11–0.26), respectively. Figure S3 gives an overview of all parameter estimates. In the absence of interventions, the estimated basic reproduction number was 1.30 (95%CrI 1.15–1.43). The analyses show that without vertical transmission the basic reproduction number would be 0.95 (95%CrI 0.80–1.12). Combining the incidence estimates of the transmission model with an estimate of 3.034 DALYs per case of cCMV yielded an estimated burden of cCMV in the Netherlands of 3527 DALYs per year (95%CrI 1397–7098) or 20.75 DALYs per 100,000 population per year (95%CrI 8.22–41.75) (see Additional file 1: Appendix).

### Vaccination dynamics

**Fig. 3:**
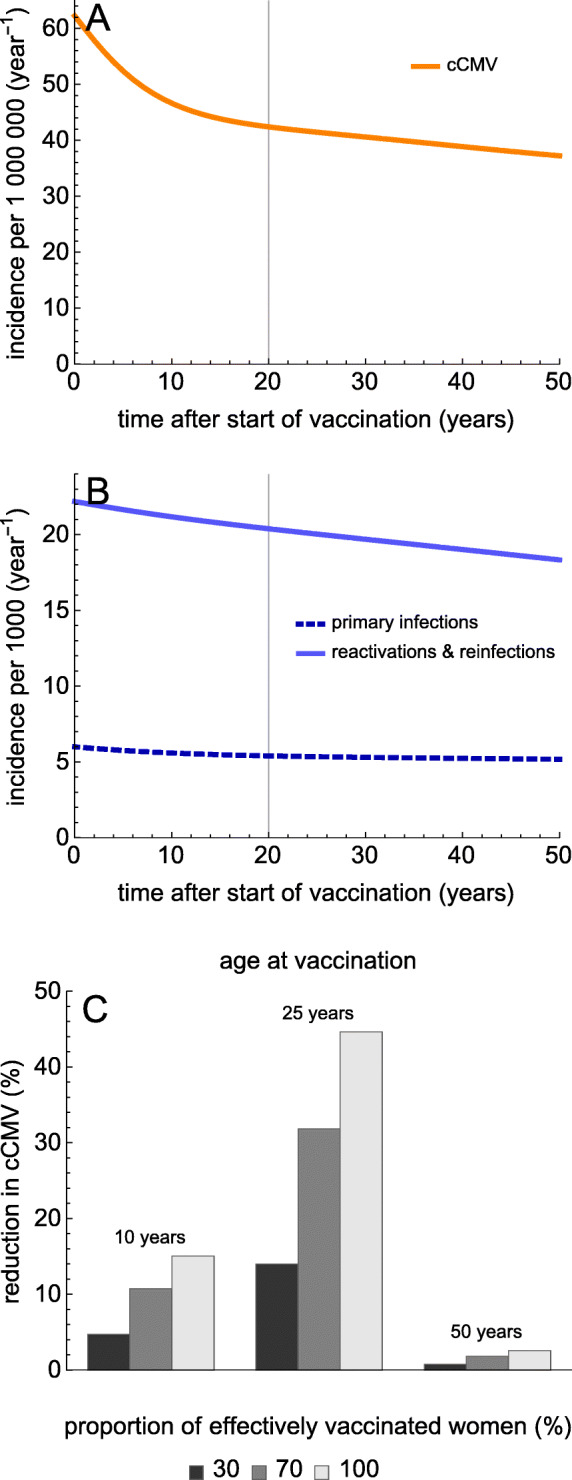
Universal vaccination of females with a vaccine preventing (re-)infection and reactivation. **a** The incidence of cCMV. **b** The incidence of primary infections and re-infections/reactivations during the first 50 years after the start of vaccination of 25-year-old women. The proportion of effectively vaccinated women (vaccination coverage × vaccine efficacy) is 70%, and the duration of protection is 10 years. **c** The reduction in cCMV after 20 years for different vaccination ages (10, 25, and 50 years) and proportions of effectively vaccinated women

All interventions are able to reduce the incidence arising through each route of transmission. The quantitative impact, however, depends strongly on the intervention (hygiene measures, vaccination during pregnancy, universal vaccination), mode of action of the vaccine (prevention of infection vs prevention of (re-)infection and reactivation), vaccination age and sex, and duration of protection by vaccination. Figure 3 shows results for vaccination of women, with a vaccine that protects for 10 years against infection and re-infection or reactivation. Here, the age at vaccination is varied from 10 to 25 years, and to 50 years, while the effectively vaccinated proportion is 30%, 70%, or 100%. Figure 3a shows that for vaccination of 25-year-old women the incidence of cCMV decreases quite rapidly during the first 10–20 years after the start of the vaccination program, from 62 cases per million per year at the start to 42 cases per million per year after 20 years. After this, the incidence of cCMV keeps decreasing due to the indirect benefits of vaccination, albeit at a much slower pace (1 case per million per 5 years). In this scenario, the incidence of primary infection and re-infection/reactivation also decrease, but at a relatively slow pace (Fig. 3b and Table 1).

**Table 1:** Impact of interventions on cCMV, primary infection, and re-infection/reactivation

Intervention scenario		Reduction %	Reduction %		
	Reduction %	incidence	incidence	Effective	
	birth prevalence	primary	re-infection/	reproduction	DALYs
	cCMV	infection	reactivation	number	prevented
	median (95%CrI)	median (95%CrI)	median (95%CrI)	median (95%CrI)	median (95%CrI)
Universal vaccination					
Prevention of (re-)infection and reactivation					
6-month-old boys and girls	5.4 (4.1–7.7)	**18.7 (16.6–21.9)**	5.2 (3.9–8.0)	1.09 (0.98–1.20)	1446 (1007–2304)
10-year-old boys and girls	11.7 (10.2–14.1)	17.2 (15.2–20.3)	7.0 (5.0–10.0)	1.10 (0.98–1.21)	3,558 (2731–4979)
10-year-old girls	10.8 (9.7–12.8)	10.5 (9.1–13.2)	4.7 (3.5–7.2)	1.13 (1.01–1.26)	3,267 (2515–4386)
25-year-old women	31.7 (30.6–33.6)	9.9 (8.6–12.5)	**7.9 (6.5–10.8)**	1.11 (0.98–1.22)	15,969 (12,560–19,993)
Prevention of infection					
6-month-old boys and girls	3.2 (2.3–4.4)	15.4 (13.9–17.7)	2.4 (1.8–3.9)	1.13 (1.01–1.24)	788 (465–1091)
10-year-old boys and girls	5.6 (4.4-6.7)	12.5 (11.0–14.3)	2.2 (1.5–3.3)	1.16 (1.04–1.28)	1584 (1063–2087)
10-year-old girls	5.3 (4.2–6.0)	7.0 (6.2–8.1)	1.5 (1.0–2.3)	1.19 (1.07–1.31)	1500 (1012–1918)
25-year-old women	8.4 (6.6–10.7)	4.5 (4.1–4.9)	1.1 (0.9–1.5)	1.25 (1.12–1.36)	4227 (2646–4968)
Vaccination during pregnancy	**71.4 (71.0–71.9)**	2.4 (0.3–4.8)	5.9 (4.3–8.5)	**1.05 (0.92–1.18)**	**49,705 (40,280–61,435)**
Hygienic measures	1.8 (1.0–2.8)	3.0 (2.5–4.0)	0.9 (0.6–1.7)	1.27 (1.13–1.38)	819 (470–1278)

The reductions are evaluated 20 years after the start of the intervention. The proportion of effectively vaccinated persons (vaccination coverage × vaccine efficacy) is 70%, and the average duration of protection is 10 years. Hygiene measures are modeled as a 70% reduction in infectious contacts between women of reproductive age (15–50 years) and young children (0–5 years). The effective reproduction number is defined as the average number of secondary infections at the start of an epidemic with one infected individual introduced in a population where 70% of persons are effectively vaccinated. This number smaller than 1 indicates that a given intervention is going to lead to the disease elimination in the long run. The burden of disease prevented by an intervention over the time period of 20 years in the Dutch population is given by the number of DALYs prevented

Figure 3c illustrates that the reduction in cCMV after 20 years also depends strongly on the age at vaccination and proportion of the population that is effectively protected after vaccination. Unsurprisingly, the reduction in cCMV increases with increasing vaccinated proportion. Further, reductions in cCMV are generally small for vaccination programs in women who are past their reproductive age (e.g., 50 years), because there are no direct effects of vaccination on cCMV and only limited indirect effects on cCMV via reduction of prevalence in the population. The highest benefits are obtained if vaccination is given at an age where vaccine protection covers the main reproductive period (i.e., 25 years). Then, there are substantial benefits, to the extent that more than 30% and 40% of cCMV can be prevented if 70% and 100% of women are effectively vaccinated, respectively. Interestingly, where vaccination of women at 25 years is more effective than vaccination at 10 years in reducing cCMV (after 20 years), the latter strategy is slightly more effective at reducing primary infections (Table 1; 10.5% vs 9.9%).

### A comparison of scenarios

Table 1 shows an overview of the impact of interventions on the reduction in the incidence of cCMV (henceforth called birth prevalence) and incidence of primary infection and re-infection/reactivation within 20 years. Intervention strategies aimed at reducing transmission from mother to child can yield substantial reductions of cCMV. Universal vaccination of females at the age of 10 and 25 years with a vaccine preventing (re-)infection and reactivation leads to 10.8% (95%CrI 9.7–12.8%) and 31.7% (95%CrI 30.6–33.6%) reduction of cCMV, respectively. If the vaccine administered to females prevents infection only, the reduction of cCMV would be 5.3% (95%CrI 4.2–6.0%) and 8.4% (95%CrI 6.6–10.7%). Vaccination of males and females at the age of 6 months, to the contrary, leads to smaller reduction in cCMV (median of 5.4%) but a much larger reduction in primary infections (median of 18.7%). Vaccination during pregnancy is very effective in reducing cCMV (median of 71.4%), but it has the least impact on primary infections. Hygiene measures are ineffective in reducing any type of infections.

**Fig. 4:**
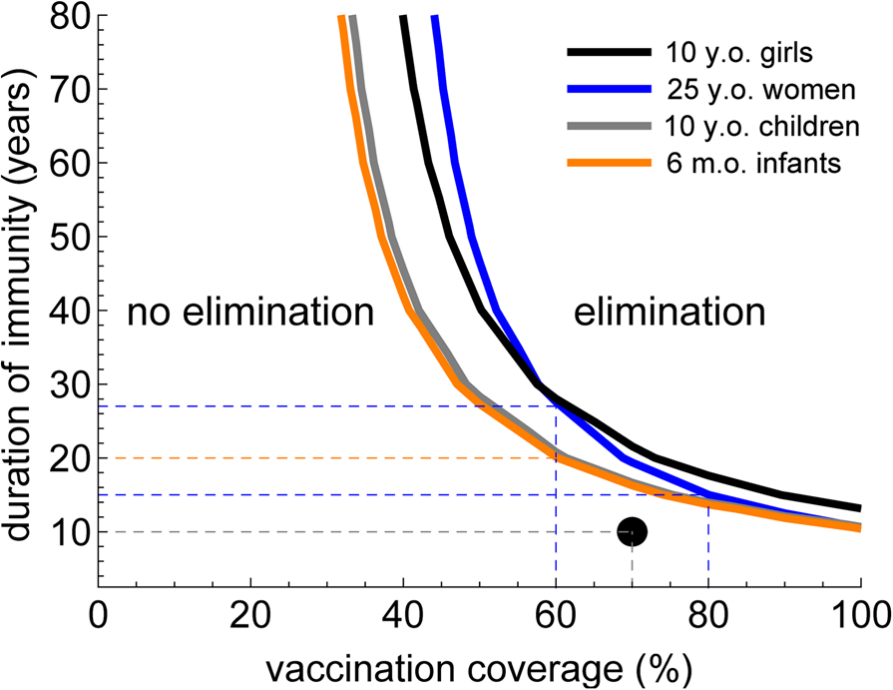
Elimination and persistence for universal vaccination with a vaccine preventing (re-)infection and reactivation. CMV elimination is feasible for effectively vaccinated proportions and durations of protection above the curve, while below the curve CMV cannot be eliminated. The curves correspond to the effective reproduction number equal to 1. The parameter values used in Table 1 are depicted as the black circle (70% and 10 years). For all vaccination strategies, the longer the duration of protection, the smaller proportion of persons would need to be effectively vaccinated to eliminate CMV. Vaccination of females only at the age of 10 or 25 years would require a vaccine with a longer duration of protection for elimination than vaccination of both males and females at the age of 6 months or at 10 years (e.g., about 27 years vs 20 years if 60% of the target group are effectively vaccinated)

If 70% of persons are effectively vaccinated and the duration of protection is 10 years, the effective reproduction numbers for all scenarios are above 1 (Table 1). This means that CMV elimination cannot be achieved. We further explored for which proportion of effectively vaccinated persons and the duration of protection CMV elimination would be feasible. Figure 4 shows the regions of persistence and elimination for the universal vaccination with a vaccine protecting against (re-)infection and reactivation. The black circle indicates parameter values used in Table 1. Our analyses show that for all vaccination strategies, the longer the duration of protection, the smaller the proportion of persons that needs to be effectively vaccinated to eliminate CMV. Vaccinated proportions and durations of protection required to achieve elimination by vaccinating males and females at the age of 6 months and by the same strategy at the age of 10 years are similar (e.g., 60% and 20 years, correspondingly). However, vaccinating effectively the same proportion of females only at the age of 10 or 25 years would require a vaccine with a longer duration of protection for elimination (i.e., 27 years for 60%).

## Discussion

Congenital CMV is a disease with serious and lifelong sequelae [3, 42]. We estimated that the burden of cCMV in the Netherlands is around 3527 DALYs per year (95%CrI: 1397–7098) or 20.75 DALYs per 100,000 persons per year (95%CrI 8.22–41.75). This burden is quite high and comparable to the burden of pertussis (3235 DALYs per year), chlamydia (3551 DALYs per year), or campylobacter (3314 DALYs per year), against which vaccination programs or other types of interventions are in place in the Netherlands [50]. Worldwide, the burden of cCMV is estimated to be even higher, as maternal prevalence of infection is higher in developing countries than in Europe and North America. Here, we have shown that several vaccination strategies have the potential to reduce the birth prevalence of cCMV in a time span of 20 years, thereby significantly reducing disease burden as measured by DALYs prevented (Table 1). Of these, vaccination during pregnancy is expected to be the most effective in preventing cCMV and its related disease burden as it specifically targets those at risk. Vaccination during pregnancy is expected to prevent almost 50,000 DALYs over a time period of 20 years, which is about 70% of all DALYs that would occur without intervention. After 20 years of vaccination, the annual disease burden from cCMV would be reduced to around 1000 DALYs per year or 6 DALYs per 100,000 population per year, which would be lower than the burden from gonorrhea (1271 DALYs per year) or invasive meningococcal disease (1065 DALYs per year) [50]. Altogether, our modeling and burden estimates before and after vaccination indicate that maternal vaccination with an effective vaccine can substantially reduce the burden of cCMV.

An alternative would be indiscriminate vaccination of women with a vaccine preventing (re-)infection and reactivation before the age at which they generally will have children (20–35 years in the Netherlands). However, in terms of burden prevented, this strategy is much less effective than vaccination of pregnant women with approximately 20,000 DALYs or 30% of the expected burden prevented over a time period of 20 years. On the other hand, a general vaccination program targeting young women has the advantage that it not only reduces cCMV in the short run (albeit less so than vaccination during pregnancy), but has a larger contribution to reducing CMV circulation in the population. This potentially has as a byproduct some added advantages, for instance by indirectly protecting solid organ and stem cell transplants, and other persons with reduced immunocompetence. In our analyses, both vaccination strategies could in the long run even lead to elimination, but this would require a sufficiently high vaccination coverage together with a long duration of protection by vaccination spanning the reproductive life span (e.g., 80% of women effectively vaccinated and 15 years of protection; Fig. 4). However, since CMV causes a lifelong infection and onward transmission is possible during reactivation, the timescale on which elimination could be achieved is very long, in the order of more than 100 years.

In our analyses, all other strategies including pediatric vaccination only prevent small proportions of the expected disease burden in the relevant short term (20 years, say). In fact, in the short term, a pediatric vaccination program is expected to be the least effective vaccination strategy in reducing cCMV but most effective at reducing primary infections. In addition, pediatric vaccination also can achieve elimination with lower effort (e.g., lower vaccinated proportion or shorter duration of protection) when compared to vaccination of young women and could be a better vaccination strategy if the goal is to reduce overall CMV transmission in the long term. This is due to the fact that the indirect effects of vaccination on horizontal transmission are strongest when the vaccine is administered at an age when contact rates are highest (i.e., children). Combining universal childhood vaccination and vaccination of pregnant women would thus seem the optimal strategy. We hope that our analyses will help to support future health economic assessments of potential CMV vaccine candidates, although our model may require modifications to support additional strategies (e.g., for other populations such as high-risk groups) and the combination of strategies (e.g., universal childhood vaccination and vaccination of pregnant women) that we did not consider here.

The feasibility of the different vaccination scenarios deserves special attention. In the comparative analyses (Table 1), we assumed that the proportion of persons that is effectively vaccinated is 70% for all scenarios. This proportion is high but could potentially be achieved for pregnant women, infants, or adolescent children if CMV vaccination is implemented in immunization programs for other diseases. For adult vaccination, much lower coverage could be expected, and the effects on cCMV will be respectively smaller. For example, if only 40% of 25-year-old women are effectively vaccinated with a vaccine preventing (re-)infection and reactivation, the birth prevalence of cCMV is reduced by 18.5%, as compared to 31.7% when the proportion of effectively vaccinated women is 70% (Table 1).

Maternal acquisition of CMV via contact with a child under 3 years of age is an important transmission route for pregnant women [25]. Currently recommended hygienic measures for reducing the risk of CMV acquisition during pregnancy center around minimizing contact with saliva and urine from young children by not sharing food, utensils, or cups with a child and also washing hands after changing diapers. The effect of hygiene intervention on CMV primary infection during pregnancy was demonstrated in controlled studies [25]. Our model, however, predicts that hygiene measures aimed to protect women of reproductive age with exposure to young children would have little impact on reducing cCMV. Decreasing infectious contacts between women and young children by 70% results only in 1.8% reduction of cCMV, because women continue to acquire CMV via contacts with older persons (e.g., older children or partners). This suggests that hygiene counseling for pregnant women should not be focused on prevention of potential infectious contacts with young children but rather with persons of all ages. In fact, in studies demonstrating the effectiveness of hygiene intervention [24, 25] women might have used hygiene precautions with a broader range of contacts, in particular, with their (seropositive) spouses who, in some studies, received detailed hygiene information as well.

Main strengths of our study are that all known transmission routes are included in one transmission model analysis and that parameters of the model have been estimated using two large unbiased population studies [33, 34]. Thus, the baseline for the analyses is consistent with the epidemiology of CMV. By evaluating a suite of vaccination scenarios, our analyses have provided, for the first time, a sound empirical basis for comparative analysis of possible interventions, among which hygiene measures and vaccination during pregnancy were considered for the first time. This comparison suggests that vaccination of pregnant women will result in a greater decline in cCMV than vaccination of infants and/or young women considered in previous modeling studies [38–40], therefore highlighting a need for the development of a vaccine that can be administered during pregnancy. Although our model was parameterized using data from the Netherlands, conclusions can be drawn also for the possible impact of vaccination in resource-limited settings, where maternal prevalence is higher. Our analysis suggests that vaccination during pregnancy could have an even higher impact on disease burden in these settings, and also hygiene measures could potentially be more effective. Vaccination and counseling could be integrated in antenatal health care, which reaches women in reproductive age in many resource-poor settings.

A number of limitations need scrutiny. First, we have restricted our statistical analyses to persons of western ethnicity. This was done to not overcomplicate an already complex transmission model. However, it is known that CMV prevalence in the Netherlands is higher in persons of non-western ethnicity [33], and how this would impact the effectiveness of vaccination is unknown. Second, estimates of the reactivation rates, infectivity of re-infection and reactivation, and probability to move from the class with low to the class with high antibody concentrations depend sensitively on the assumed prior distributions of the reactivation rates. The sensitivity analyses for prior distributions for these rates demonstrate that the impact of vaccination, in particular the estimated reductions in cCMV, primary infections, re-infections, and reactivations are quantitatively very similar to the results in the main text (Table S2). Hence, while considerable uncertainty surrounds the actual magnitude of the reactivation rates and associated parameters, the impact on the effectiveness of vaccination is found to be small.

## Conclusions

In conclusion, our study has provided a comparative analysis of the expected effectiveness of vaccination against CMV and has shown that several options exist to reduce the incidence of congenital CMV in a time span of 20 years. Depending on the proportion of the target group that is effectively vaccinated and the duration of protection after vaccination, significant long-term reductions in the overall circulation of CMV at the population level also seem within reach. It is even conceivable that a vaccination campaign with an effective vaccine and high vaccination coverage (>80*%*) could in the long run lead to elimination. In view of the associations of CMV infection with accelerated aging of the immune system [8, 51, 52], such long-term benefits could in the long run factor in heavily in future analyses of the effectiveness and cost-effectiveness of CMV vaccination.

## Supplementary information

**Additional file 1** Appendix: This appendix contains all mathematical details of the analyses presented in the main text.

## Abbreviations

cCMV: Congenital cytomegalovirus infection
CMV: Cytomegalovirus
DALYs: Disability-adjusted life years
