# Supplementary material for: Short- and long-term impact of vaccination against cytomegalovirus: a modeling study
Source: BMC Med. 2020 Jul 2;18:174. doi: 10.1186/s12916-020-01629-3 (PMC7331215; doi:10.1186/s12916-020-01629-3)
Supplement: Supplementary file 1 — Additional file 1 Appendix: This appendix contains all mathematical details of the analyses presented in the main text. [file 12916_2020_1629_MOESM1_ESM.pdf]

Appendix
Short- and long-term impact of vaccination against cytomegalovirus: a modeling study

Ganna Rozhnova[1], *Mirjam E. Kretzschmar, Fiona van der Klis, Debbie van Baarle, Marjolein Korndewal, Ann C. Vossen, Michiel van Boven*

Contents

1 Continuous model formulation **2**

2 Model with discrete age groups **4**

3 Force of infection **7**

4 Simplified model with discrete age groups **8**

5 Calculation of the effective reproduction number **12**

6 Model with universal vaccination **13**

6.1 Prevention of infection . . . 14

6.1.1 6-months-old boys and girls and life-long protection . . . 14

6.1.2 6-months-old boys and girls and waning protection . . . 17

6.1.3 Individuals in age class k' and waning protection . . . 18

6.2 Prevention of (re-)infection and reactivation . . . 20

6.2.1 Individuals in age class k' and waning protection . . . 20

7 Model outcomes **24**

8 Parameter summary **25**

9 Parameter inference **25**

10 Results for the scenario with high reactivation rates **27**

11 DALY computation **31**

[1]corresponding author: G.Rozhnova@umcutrecht.nl

1 Continuous model formulation

Let us denote by $S^i(a,t)$ the age density of seronegative (susceptible, S) individuals of sex $i \in \{♂,♀\}$ at time t. Similarly we denote by $L^i(a,t)$ and $B^i(a,t)$ the age densities of latently infected (latent, L) with low antibody concentrations and latently infected with high antibody concentrations (boosted, B) of sex i at time t. The age densities of infectious individuals with primary acute infection (I_1), and re-infection or reactivation from the L and B classes (I_2 and I_3) are $I_1^i(a,t)$, $I_2^i(a,t)$ and $I_3^i(a,t)$, respectively.

The age density of individuals of sex i at time t is given by

$$P^i(a,t) = S^i(a,t) + L^i(a,t) + B^i(a,t) + I_1^i(a,t) + I_2^i(a,t) + I_3^i(a,t). \tag{1}$$

The total size of the population of sex i at time t is obtained by integrating $P^i(a,t)$ defined by Eq. (1) over all ages

$$N^i(t) = \int_0^M P^i(a,t)\mathrm{d}a, \tag{2}$$

where M is the maximum attainable age of the population of sex i.

The age-structured CMV model can then be formulated as follows

$$
\begin{aligned}
\frac{\partial S^i(a,t)}{\partial t} + \frac{\partial S^i(a,t)}{\partial a} &= -\lambda^i(a,t)S^i(a,t) - \mu^i(a)S^i(a,t), && (3)\\
\frac{\partial I_1^i(a,t)}{\partial t} + \frac{\partial I_1^i(a,t)}{\partial a} &= \lambda^i(a,t)S^i(a,t) - \left[\gamma_1^i(a) + \mu^i(a)\right] I_1^i(a,t), && (4)\\
\frac{\partial L^i(a,t)}{\partial t} + \frac{\partial L^i(a,t)}{\partial a} &= \gamma_1^i(a)I_1^i(a,t) - \left[\rho^i(a) + z\lambda^i(a,t)\right] L^i(a,t) - \mu^i(a)L^i(a,t) + \\
&+ (1-p_{LB})\gamma_2^i(a)I_2^i(a,t), && (5)\\
\frac{\partial I_2^i(a,t)}{\partial t} + \frac{\partial I_2^i(a,t)}{\partial a} &= \left[\rho^i(a) + z\lambda^i(a,t)\right] L^i(a,t) - \left[\gamma_2^i(a) + \mu^i(a)\right] I_2^i(a,t), && (6)\\
\frac{\partial B^i(a,t)}{\partial t} + \frac{\partial B^i(a,t)}{\partial a} &= p_{LB}\gamma_2^i(a)I_2^i(a,t) - \left[\rho^i(a) + z\lambda^i(a,t)\right] B^i(a,t) - \mu^i(a)B^i(a,t) + \\
&+ \gamma_3^i(a)I_3^i(a,t), && (7)\\
\frac{\partial I_3^i(a,t)}{\partial t} + \frac{\partial I_3^i(a,t)}{\partial a} &= \left[\rho^i(a) + z\lambda^i(a,t)\right] B^i(a,t) - \left[\gamma_3^i(a) + \mu^i(a)\right] I_3^i(a,t). && (8)
\end{aligned}
$$

The initial and the boundary conditions are

$$S^i(0,t) = \int_0^M \phi^i(a)\{S^{♀}(a,t) + (1-q)[I_1^{♀}(a,t) + L^{♀}(a,t) + I_2^{♀}(a,t) + B^{♀}(a,t) + I_3^{♀}(a,t)]\}\mathrm{d}a, \tag{9}$$

$$I_1^i(0,t) = q\int_0^M \phi^i(a)[I_1^{♀}(a,t) + L^{♀}(a,t) + I_2^{♀}(a,t) + B^{♀}(a,t) + I_3^{♀}(a,t)]\mathrm{d}a, \tag{10}$$

$$L^i(0,t) = 0, \tag{11}$$

$$I_2^i(0,t) = 0, \tag{12}$$

$$B^i(0,t) = 0, \tag{13}$$

$$I_3^i(0,t) = 0, \tag{14}$$

$$S^i(a,0) = S_0^i(a), \tag{15}$$

$$I_1^i(a,0) = I_{10}^i(a), \tag{16}$$

$$L^i(a,0) = L_0^i(a), \tag{17}$$

$$I_2^i(a,0) = I_{20}^i(a), \tag{18}$$

$$B^i(a,0) = B_0^i(a), \tag{19}$$

$$I_3^i(a,0) = I_{30}^i(a). \tag{20}$$

In the model defined by Eqs. (3)-(20) $\mu^i(a)$ denotes the age-specific death rate of individuals of sex i; $\phi^i(a)$ is the age-specific fertility rate of women; $q \in [0,1]$ is the probability of vertical transmission; $\lambda^i(a,t)$ is the force of infection for individuals of sex i and age a at time t; $\rho^i(a)$ is age-specific reactivation rate for individuals of sex i; $z \in [0,1]$ is the reduction in susceptibility to re-infection in latently infected individuals compared to seronegative individuals; $p_{LB} \in [0,1]$ is the probability of progression from low to high antibody concentrations; $\gamma_1^i(a)$, $\gamma_2^i(a)$, and $\gamma_3^i(a)$ are the rates of progression from acute infectious state to latent uninfectious state.

The dynamics of the population can be determined independently from the CMV dynamics. The equation for the age density of individuals of sex i at time t is obtained by adding Eqs. (3)-(8)

$$\frac{\partial P^i(a,t)}{\partial t} + \frac{\partial P^i(a,t)}{\partial a} = -\mu^i(a)P^i(a,t). \tag{21}$$

Adding up the boundary, Eqs. (9)-(14), and initial, Eqs. (15)-(20), conditions we obtain

$$P^i(0,t) = \int_0^M \phi^i(a) P^{♀}(a,t)\mathrm{d}a, \quad (22)$$

$$P^i(a,0) = P_0^i(a), \quad (23)$$

where $P_0^i(a) = S_0^i(a) + L_0^i(a) + B_0^i(a) + I_{10}^i(a) + I_{20}^i(a) + I_{30}^i(a)$.

In the demographic steady state the reproduction rate of the population is 1, which can be expressed as follows

$$\int_0^M \phi^i(a) l^i(a)\mathrm{d}a = 1, \quad (24)$$

where $l^i(a)$ is the probability of individuals of sex i surviving till age a

$$l^i(a) = \exp\left(-\int_0^a \mu^i(a')\mathrm{d}a'\right). \quad (25)$$

2 Model with discrete age groups

In this section we derive from the initial boundary-value problem defined by Eqs. (3)-(20) a system of $6 \times 2 \times n$ (# of variables × # of sexes × # of age groups) ordinary differential equations for the number of individuals of different types at time t in n age groups. The age groups are defined by age intervals $[a_{k-1}, a_k]$ with $a_0 = 0 < a_1 < a_2 \ldots < a_{n-1} < a_n = M$, $k = 1, \ldots, n$.

Let us denote by $S_k^i(t)$, $L_k^i(t)$, $B_k^i(t)$, $I_{1,k}^i$, $I_{2,k}^i$ and $I_{3,k}^i$ the number of S, L, B, I_1, I_2, and I_3 individuals of sex $i \in \{♂,♀\}$ in the k-th age group $[a_{k-1}, a_k]$ as follows

$$S_k^i(t) = \int\limits_{a_{k-1}}^{a_k} S^i(a,t)\mathrm{d}a, \tag{26}$$

$$L_k^i(t) = \int\limits_{a_{k-1}}^{a_k} L^i(a,t)\mathrm{d}a, \tag{27}$$

$$B_k^i(t) = \int\limits_{a_{k-1}}^{a_k} B^i(a,t)\mathrm{d}a, \tag{28}$$

$$I_{1,k}^i(t) = \int\limits_{a_{k-1}}^{a_k} I_1^i(a,t)\mathrm{d}a, \tag{29}$$

$$I_{2,k}^i(t) = \int\limits_{a_{k-1}}^{a_k} I_2^i(a,t)\mathrm{d}a, \tag{30}$$

$$I_{3,k}^i(t) = \int\limits_{a_{k-1}}^{a_k} I_3^i(a,t)\mathrm{d}a. \tag{31}$$

We assume that the rates of fertility, death, reactivation, progression from acute to latent state and the force of infection are constant in each age group, i. e.

$$\phi^i(a) = \phi_k^i, \tag{32}$$
$$\mu^i(a) = \mu_k^i, \tag{33}$$
$$\rho^i(a) = \rho_k^i, \tag{34}$$
$$\gamma_1^i(a) = \gamma_{1,k}^i, \tag{35}$$
$$\gamma_2^i(a) = \gamma_{2,k}^i, \tag{36}$$
$$\gamma_3^i(a) = \gamma_{3,k}^i, \tag{37}$$
$$\lambda^i(a,t) = \lambda_k^i(t) \tag{38}$$

for a in the age group $[a_{k-1}, a_k]$, $k = 1, \ldots, n$.

We integrate the equation for $S^i(a,t)$, Eq. (3), from $a_0 = 0$ to a_1

$$\int\limits_{a_0=0}^{a_1} \frac{\partial S^i(a,t)}{\partial t}\mathrm{d}a + \int\limits_{a_0=0}^{a_1} \frac{\partial S^i(a,t)}{\partial a}\mathrm{d}a = -\int\limits_{a_0=0}^{a_1} \lambda^i(a,t)S^i(a,t)\mathrm{d}a - \int\limits_{a_0=0}^{a_1} \mu^i(a)S^i(a,t)\mathrm{d}a. \tag{39}$$

Using Eqs. (26), (33) and (38) the above equation can be rewritten in the following form

$$\frac{\mathrm{d}S_1^i(t)}{\mathrm{d}t} + S^i(a_1,t) - S^i(0,t) = -\lambda_1^i(t)S_1^i(t) - \mu_1^i S_1^i(t). \tag{40}$$

Using Eqs. (26)-(32) the boundary condition given by Eq. (9) becomes

$$S^i(0,t) = \sum_{k=1}^{n} \phi_k^i \{S_k^{♀}(t) + (1-q)[L_k^{♀}(t) + B_k^{♀}(t) + I_{1,k}^{♀}(t) + I_{2,k}^{♀}(t) + I_{3,k}^{♀}(t)]\}. \tag{41}$$

We further define the progression rate from age group k to age group $(k+1)$ as follows

$$m_k = \frac{S^i(a_k,t)}{S_k^i(t)}. \tag{42}$$

Finally, the equation for the number of susceptible individuals of sex i in the first age group $[0, a_1]$ becomes

$$\begin{aligned} \frac{\mathrm{d}S_1^i(t)}{\mathrm{d}t} &= \sum_{k=1}^{n} \phi_k^i \{S_k^{♀}(t) + (1-q)[L_k^{♀}(t) + B_k^{♀}(t) + I_{1,k}^{♀}(t) + I_{2,k}^{♀}(t) + I_{3,k}^{♀}(t)]\} - \\ &- m_1 S_1^i(t) - \lambda_1^i(t)S_1^i(t) - \mu_1^i S_1^i(t). \end{aligned} \tag{43}$$

Integrating Eq. (3) from a_{k-1} to a_k, where $k = 2, \ldots, n$, we obtain

$$\int\limits_{a_{k-1}}^{a_k} \frac{\partial S^i(a,t)}{\partial t}\mathrm{d}a + \int\limits_{a_{k-1}}^{a_k} \frac{\partial S^i(a,t)}{\partial a}\mathrm{d}a = -\int\limits_{a_{k-1}}^{a_k} \lambda^i(a,t)S^i(a,t)\mathrm{d}a - \int\limits_{a_{k-1}}^{a_k} \mu^i(a)S^i(a,t)\mathrm{d}a. \tag{44}$$

The above equation simplifies to

$$\frac{\mathrm{d}S_k^i(t)}{\mathrm{d}t} + S^i(a_k,t) - S^i(a_{k-1},t) = -\lambda_k^i(t)S_k^i(t) - \mu_k^i S_k^i(t). \tag{45}$$

Using Eq. (42) we finally arrive to the following equation

$$\frac{\mathrm{d}S_k^i(t)}{\mathrm{d}t} = m_{k-1}S_{k-1}^i(t) - m_k S_k^i(t) - \lambda_k^i(t)S_k^i(t) - \mu_k^i S_k^i(t), \tag{46}$$

where $k = 2, \ldots, n$.

The equations for other variables can be obtained similarly. The final system of the ordinary differential equations describing the model with discrete age groups reads as follows

$$
\begin{aligned}
\frac{\mathrm{d}S_1^i(t)}{\mathrm{d}t} &= \sum_{k=1}^{n} \phi_k^i \{S_k^{♀}(t) + (1-q)[I_{1,k}^{♀}(t) + L_k^{♀}(t) + I_{2,k}^{♀}(t) + B_k^{♀}(t) + I_{3,k}^{♀}(t)]\} - \\
&- \left[m_1 + \lambda_1^i(t) + \mu_1^i\right] S_1^i(t), && (47)\\
\frac{\mathrm{d}S_k^i(t)}{\mathrm{d}t} &= m_{k-1} S_{k-1}^i(t) - \left[m_k + \lambda_k^i(t) + \mu_k^i\right] S_k^i(t), && (48)\\
\frac{\mathrm{d}I_{1,1}^i(t)}{\mathrm{d}t} &= q \sum_{k=1}^{n} \phi_k^i [I_{1,k}^{♀}(t) + L_k^{♀}(t) + I_{2,k}^{♀}(t) + B_k^{♀}(t) + I_{3,k}^{♀}(t)] + \lambda_1^i(t) S_1^i(t) - \\
&- \left[m_1 + \gamma_{1,1}^i + \mu_1^i\right] I_{1,1}^i(t), && (49)\\
\frac{\mathrm{d}I_{1,k}^i(t)}{\mathrm{d}t} &= \lambda_k^i(t) S_k^i(t) + m_{k-1} I_{1,k-1}^i(t) - \left[m_k + \gamma_{1,k}^i + \mu_k^i\right] I_{1,k}^i(t), && (50)\\
\frac{\mathrm{d}L_1^i(t)}{\mathrm{d}t} &= \gamma_{1,1}^i I_{1,1}^i(t) - \left[m_1 + \rho_1^i + z\lambda_1^i(t) + \mu_1^i\right] L_1^i(t) + (1-p_{LB})\gamma_{2,1}^i I_{2,1}^i(t), && (51)\\
\frac{\mathrm{d}L_k^i(t)}{\mathrm{d}t} &= \gamma_{1,k}^i I_{1,k}^i(t) + m_{k-1} L_{k-1}^i(t) - \left[m_k + \rho_k^i + z\lambda_k^i(t) + \mu_k^i\right] L_k^i(t) + \\
&+ (1-p_{LB})\gamma_{2,k}^i I_{2,k}^i(t), && (52)\\
\frac{\mathrm{d}I_{2,1}^i(t)}{\mathrm{d}t} &= \left[\rho_1^i + z\lambda_1^i(t)\right] L_1^i(t) - \left[m_1 + \gamma_{2,1}^i + \mu_1^i\right] I_{2,1}^i(t), && (53)\\
\frac{\mathrm{d}I_{2,k}^i(t)}{\mathrm{d}t} &= \left[\rho_k^i + z\lambda_k^i(t)\right] L_k^i(t) + m_{k-1} I_{2,k-1}^i(t) - \left[m_k + \gamma_{2,k}^i + \mu_k^i\right] I_{2,k}^i(t), && (54)\\
\frac{\mathrm{d}B_1^i(t)}{\mathrm{d}t} &= p_{LB}\gamma_{2,1}^i I_{2,1}^i(t) - \left[m_1 + \rho_1^i + z\lambda_1^i(t) + \mu_1^i\right] B_1^i(t) + \gamma_{3,1}^i I_{3,1}^i(t), && (55)\\
\frac{\mathrm{d}B_k^i(t)}{\mathrm{d}t} &= p_{LB}\gamma_{2,k}^i I_{2,k}^i(t) + m_{k-1} B_{k-1}^i(t) - \left[m_k + \rho_k^i + z\lambda_k^i(t) + \mu_k^i\right] B_k^i(t) + \\
&+ \gamma_{3,k}^i I_{3,k}^i(t), && (56)\\
\frac{\mathrm{d}I_{3,1}^i(t)}{\mathrm{d}t} &= \left[\rho_1^i + z\lambda_1^i(t)\right] B_1^i(t) - \left[m_1 + \gamma_{3,1}^i + \mu_1^i\right] I_{3,1}^i(t), && (57)\\
\frac{\mathrm{d}I_{3,k}^i(t)}{\mathrm{d}t} &= \left[\rho_k^i + z\lambda_k^i(t)\right] B_k^i(t) + m_{k-1} I_{3,k-1}^i(t) - \left[m_k + \gamma_{3,k}^i + \mu_k^i\right] I_{3,k}^i(t), && (58)
\end{aligned}
$$

where $k = 2, \ldots, n$.

3 Force of infection

The force of infection for individuals of sex i and age a at time t is expressed as follows

$$
\lambda^i(a,t) = \sum_{j \in \{♂,♀\}} \int_0^M c^{ij}(a,a') \left[\beta_1 \frac{I_1^j(a',t)}{P^j(a',t)} + \beta_2 \frac{I_2^j(a',t)}{P^j(a',t)} + \beta_2 \frac{I_3^j(a',t)}{P^j(a',t)}\right] \mathrm{d}a', \qquad (59)
$$

where β_1 and β_2 are proportionality parameters determining the infectivities of primary infection and re-infection/reactivation, $c^{ij}(a,a')$ is the contact rate between individuals of

sex i and age a and individuals of sex j and age a'.

More precisely, $c^{ij}(a,a')$ represents the number of contacts per unit of time that one individual of sex i and age a receives from individuals of sex j and age a'. The contact rate $c^{ij}(a,a')$ can thus be written down as the product of the number of contacts per unit of time between one individual of sex i and age a and one individual of sex j and age a' and the age density of individuals of sex j at time t

$$c^{ij}(a,a') = \tilde{c}^{ij}(a,a')P^{j}(a',t). \tag{60}$$

Note that by definition $\tilde{c}^{ij}(a,a')$ is symmetric while $c^{ij}(a,a')$ is not. Using the above equation the expression for the force of infection simplifies to

$$\lambda^{i}(a,t) = \sum_{j\in\{♂,♀\}} \int_{0}^{M} \tilde{c}^{ij}(a,a')\left[\beta_1 I_1^{j}(a',t) + \beta_2 I_2^{j}(a',t) + \beta_2 I_3^{j}(a',t)\right] \mathrm{d}a'. \tag{61}$$

Eq. (61) is used in the continuous formulation of the model defined by Eqs. (3)-(8). In the model with discrete age groups defined by Eqs. (47)-(58), the force of infection has to be discretized. This is done assuming that the contact rate is constant for the interactions between age groups

$$\tilde{c}^{ij}(a,a') = \tilde{c}^{ij}_{kl} \tag{62}$$

for $a \in [a_{k-1}, a_k]$ and $a' \in [a_{l-1}, a_l]$, $k,l = 1,\dots,n$.

The force of infection for individuals of sex i in the k-th age group $[a_{k-1}, a_k]$ becomes

$$\lambda^{i}_{k}(t) = \sum_{j\in\{♂,♀\}} \sum_{l=1}^{n} \tilde{c}^{ij}_{kl}\left[\beta_1 I^{j}_{1,l}(t) + \beta_2 I^{j}_{2,l}(t) + \beta_2 I^{j}_{3,l}(t)\right](a_l - a_{l-1}). \tag{63}$$

Eq. (63) is used in the model with discrete age groups, Eqs. (47)-(58).

4 Simplified model with discrete age groups

We now aim to obtain a simplified version of the model with discrete age groups, Eqs. (47)-(58) that can be fit to the cross-sectional serological data providing information on the infection status of the Dutch population in 2006/2007, and a retrospective cohort study carried out in 2008 providing information on the birth prevalence of cCMV (i.e. the fraction of infants infected during pregnancy) [33,34]. Firstly, we assume that the male to female ratio is 1 to 1. Secondly, the population is assumed to be in the demographic equilibrium, i.e. population sizes of different age groups are constant. Finally, we model

Type I mortality, namely the probability of surviving is 1 till the maximum age M and zero above that.

We start by obtaining equations for the number of individuals of sex i in the k-th age group $[a_{k-1}, a_k]$

$$N_k^i(t) = \int\limits_{a_{k-1}}^{a_k} P^i(a,t)\mathrm{d}a = S_k^i(t) + L_k^i(t) + B_k^i(t) + I_{1,k}^i(t) + I_{2,k}^i(t) + I_{3,k}^i(t), \quad k = 1, \ldots, n. \tag{64}$$

We obtain for $N_1^i(t)$ and $N_k^i(t)$

$$\frac{\mathrm{d}N_1^i(t)}{\mathrm{d}t} = \sum_{k=1}^{n} \phi_k^i N_k^{\female}(t) - (m_1 + \mu_1^i)N_1^i(t), \tag{65}$$

$$\frac{\mathrm{d}N_k^i(t)}{\mathrm{d}t} = m_{k-1}N_{k-1}^i(t) - (m_k + \mu_k^i)N_k^i(t), \tag{66}$$

where $k = 2, \ldots, n$.

We now find the expression for the progression rate m_k from age group k to age group $(k+1)$, $k = 1, \ldots, n$ for the demographically stable population: $P^i(a,t) \equiv P^i(a)$. For this special case Eq. (21) simplifies to

$$\frac{\mathrm{d}P^i(a)}{\mathrm{d}a} = -\mu^i(a)P^i(a). \tag{67}$$

Solving the above equation on the interval $[a_{k-1}, a_k]$ results in

$$P^i(a) = P^i(a_{k-1}) \exp\left[-\mu_k^i(a - a_{k-1})\right]. \tag{68}$$

Using this expression we further integrate $P^i(a)$ over the interval $[a_{k-1}, a_k]$ to get

$$N_k^i = \int\limits_{a_{k-1}}^{a_k} P^i(a)\mathrm{d}a \tag{69}$$

$$= \int\limits_{a_{k-1}}^{a_k} P^i(a_{k-1}) \exp\left[-\mu_k^i(a - a_{k-1})\right] \mathrm{d}a \tag{70}$$

$$= \frac{P^i(a_{k-1})}{\mu_k^i} \left\{1 - \exp[-\mu_k^i(a_k - a_{k-1})]\right\}. \tag{71}$$

The progression rate m_k from age group k to age group $(k+1)$ then becomes

$$m_k \equiv \frac{P^i(a_k)}{N_k^i} = \frac{P^i(a_{k-1}) \exp\left[-\mu_k^i(a_k - a_{k-1})\right] \mu_k^i}{P^i(a_{k-1}) \left\{1 - \exp[-\mu_k^i(a_k - a_{k-1})]\right\}}. \tag{72}$$

The final expression for m_k is

$$m_k = \frac{\mu_k^i}{\exp[\mu_k^i(a_k - a_{k-1})] - 1}, \quad k = 1, \ldots, n. \tag{73}$$

Note that Type I mortality implies that all $\mu_k^i = 0$, therefore we must find the limit of m_k when $\mu_k^i \to 0$:

$$\lim_{\mu_k^i \to 0} m_k = \frac{1}{a_k - a_{k-1}}, \quad k = 1, \ldots, n. \tag{74}$$

Finally, we substitute $\mu_k^i = 0$ into Eqs. (65)-(66) and set $\mathrm{d}N_k^i(t)/\mathrm{d}t = 0$, $k = 1, \ldots, n$. We obtain the following system of equations

$$\sum_{k=1}^{n} \phi_k^i N_k^{♀} = m_1 N_1^i, \tag{75}$$

$$m_1 N_1^i = m_2 N_2^i, \tag{76}$$

$$m_2 N_2^i = m_3 N_3^i, \tag{77}$$

$$\ldots$$

$$m_{k-1} N_{k-1}^i = m_k N_k^i, \tag{78}$$

where $k = 3, \ldots, n$.

Solving Eqs. (76)-(78) we obtain the equation for N_k^i in terms of N_1^i:

$$N_k^i = \frac{m_1}{m_k} N_1^i, \quad k = 1, \ldots, n. \tag{79}$$

Since the ratio of men to women is 1 to 1, we can write Eq. (75) as follows

$$\sum_{k=1}^{n} \phi_k^i N_k^i = m_1 N_1^i. \tag{80}$$

Combining the last two expressions we obtain

$$\sum_{k=1}^{n} \phi_k^i \frac{m_1}{m_k} N_1^i = m_1 N_1^i, \tag{81}$$

which can be written as

$$\sum_{k=1}^{n} \frac{\phi_k^i}{m_k} = 1 \quad \text{or} \quad \sum_{k=1}^{n} \phi_k^i (a_k - a_{k-1}) = 1. \tag{82}$$

The above equation is the discrete version of Eq. (24). Denoting the total population size for sex i as N^i and using Eq. (79) we get

$$N^i = \sum_{k=1}^{n} N_k^i = N_1^i m_1 \sum_{k=1}^{n} \frac{1}{m_k} = N_1^i \frac{M}{a_1}. \tag{83}$$

If the population size of sex i, N^i, the maximum attainable age, M, and the age group intervals $[a_{k-1}, a_k]$ are given, the population sizes in different age groups are then determined as

$$N_k^i = \frac{N^i}{m_k \sum_{k=1}^{n} \frac{1}{m_k}} = N^i \frac{(a_k - a_{k-1})}{M}, \quad k = 1, \ldots, n. \tag{84}$$

This means that the age distribution is uniform on the interval $[a_0 = 0, a_n = M]$.

Since by construction the population sizes of different age groups do not change we can express $S_k^i(t)$ as $S_k^i(t) = N_k^i - I_{1,k}^i(t) - L_k^i(t) - I_{2,k}^i(t) - B_k^i(t) - I_{3,k}^i(t)$ and drop the equations for $S_k^i(t)$. The equations for the model with discrete age groups are

$$\begin{aligned}
\frac{\mathrm{d}I_{1,1}^i(t)}{\mathrm{d}t} &= q \sum_{k=1}^{n} \phi_k^i [I_{1,k}^{♀}(t) + L_k^{♀}(t) + I_{2,k}^{♀}(t) + B_k^{♀}(t) + I_{3,k}^{♀}(t)] - \left[m_1 + \gamma_{1,1}^i\right] I_{1,1}^i(t) + \\
&+ \lambda_1^i(t)[N_1^i - I_{1,1}^i(t) - L_1^i(t) - I_{2,1}^i(t) - B_1^i(t) - I_{3,1}^i(t)], && (85) \\
\frac{\mathrm{d}I_{1,k}^i(t)}{\mathrm{d}t} &= \lambda_k^i(t)[N_k^i - I_{1,k}^i(t) - L_k^i(t) - I_{2,k}^i(t) - B_k^i(t) - I_{3,k}^i(t)] + \\
&+ m_{k-1} I_{1,k-1}^i(t) - \left[m_k + \gamma_{1,k}^i\right] I_{1,k}^i(t), && (86) \\
\frac{\mathrm{d}L_1^i(t)}{\mathrm{d}t} &= \gamma_{1,1}^i I_{1,1}^i(t) - \left[m_1 + \rho_1^i + z\lambda_1^i(t)\right] L_1^i(t) + (1 - p_{LB})\gamma_{2,1}^i I_{2,1}^i(t), && (87) \\
\frac{\mathrm{d}L_k^i(t)}{\mathrm{d}t} &= \gamma_{1,k}^i I_{1,k}^i(t) + m_{k-1} L_{k-1}^i(t) - \left[m_k + \rho_k^i + z\lambda_k^i(t)\right] L_k^i(t) + \\
&+ (1 - p_{LB})\gamma_{2,k}^i I_{2,k}^i(t), && (88) \\
\frac{\mathrm{d}I_{2,1}^i(t)}{\mathrm{d}t} &= \left[\rho_1^i + z\lambda_1^i(t)\right] L_1^i(t) - \left[m_1 + \gamma_{2,1}^i\right] I_{2,1}^i(t), && (89) \\
\frac{\mathrm{d}I_{2,k}^i(t)}{\mathrm{d}t} &= \left[\rho_k^i + z\lambda_k^i(t)\right] L_k^i(t) + m_{k-1} I_{2,k-1}^i(t) - \left[m_k + \gamma_{2,k}^i\right] I_{2,k}^i(t), && (90) \\
\frac{\mathrm{d}B_1^i(t)}{\mathrm{d}t} &= p_{LB}\gamma_{2,1}^i I_{2,1}^i(t) - \left[m_1 + \rho_1^i + z\lambda_1^i(t)\right] B_1^i(t) + \gamma_{3,1}^i I_{3,1}^i(t), && (91) \\
\frac{\mathrm{d}B_k^i(t)}{\mathrm{d}t} &= p_{LB}\gamma_{2,k}^i I_{2,k}^i(t) + m_{k-1} B_{k-1}^i(t) - \left[m_k + \rho_k^i + z\lambda_k^i(t)\right] B_k^i(t) + \\
&+ \gamma_{3,k}^i I_{3,k}^i(t), && (92) \\
\frac{\mathrm{d}I_{3,1}^i(t)}{\mathrm{d}t} &= \left[\rho_1^i + z\lambda_1^i(t)\right] B_1^i(t) - \left[m_1 + \gamma_{3,1}^i\right] I_{3,1}^i(t), && (93) \\
\frac{\mathrm{d}I_{3,k}^i(t)}{\mathrm{d}t} &= \left[\rho_k^i + z\lambda_k^i(t)\right] B_k^i(t) + m_{k-1} I_{3,k-1}^i(t) - \left[m_k + \gamma_{3,k}^i\right] I_{3,k}^i(t), && (94)
\end{aligned}$$

where $k = 2, \ldots, n$.

In the above equations

$$N_k^i = N^i \frac{(a_k - a_{k-1})}{M} \quad \text{and} \quad m_k = \frac{1}{a_k - a_{k-1}}, \quad k = 1, \ldots, n, \tag{95}$$

and

$$\lambda_k^i(t) = \sum_{j \in \{♂,♀\}} \sum_{l=1}^{n} \tilde{c}_{kl}^{ij} \left[\beta_1 I_{1,l}^j(t) + \beta_2 I_{2,l}^j(t) + \beta_3 I_{3,l}^j(t)\right] (a_l - a_{l-1}), \quad k = 1, \ldots, n. \tag{96}$$

The steady state solutions of the model are obtained by setting the left hand sides of Eqs. (85)-(94) to zero. To calculate the disease free steady state we substitute in the resulting set of equations $q = 0$, $\lambda_k^i(t) = 0$ and $L_k^i(t) = B_k^i(t) = I_{1,k}^i(t) = I_{2,k}^i(t) = I_{3,k}^i(t) = 0$, where $i \in \{♀, ♂\}$ and $k = 1, \ldots, n$. The disease free equilibrium is

$$\bar{S}_1^i = \frac{1}{m_1} \sum_{k=1}^{n} \phi_k^i N_k^{♀}, \tag{97}$$

$$\bar{S}_k^i = \frac{m_{k-1}}{m_k} \bar{S}_{k-1}^i, \quad k = 2, \ldots, n, \tag{98}$$

$$\bar{I}_{1,k}^i = \bar{I}_{2,k}^i = \bar{I}_{3,k}^i = \bar{L}_k^i = \bar{B}_k^i = 0, \quad k = 1, \ldots, n, \tag{99}$$

where the bar denotes the steady-state solution.

5 Calculation of the effective reproduction number

In this section we describe how to calculate the basic reproduction number, R_0, in the absence of vaccination and the effective reproduction number, R_e, in the population with vaccination or hygienic intervention. We used the method known in the literature as the next-generation matrix approach. The detailed description of the method can be found in [48,49].

The steps in the calculation of R_0 for the CMV model without vaccination are

1. Calculate the Jacobian matrix, $\mathbf{J}$, of Eqs. (85)-(94).
2. Evaluate the Jacobian, $\mathbf{J}$, at the infection free equilibrium Eqs. (97)-(99).
3. Write down the Jacobian, $\mathbf{J}$, as a sum of two matrices, the matrix of transmissions $\mathbf{T}$ and the matrix of transitions $\mathbf{\Sigma}$: $\mathbf{J} = \mathbf{T} + \mathbf{\Sigma}$. The matrix of transmissions, $\mathbf{T}$, contains elements of $\mathbf{J}$ proportional to q, β_1 and β_2. The matrix of transitions, $\mathbf{\Sigma}$, contains the remaining elements of $\mathbf{J}$.

4. R_0 then equals the dominant eigenvalue of the next generation matrix defined as follows $\mathbf{K} = -\mathbf{T}\mathbf{\Sigma}^{-1}$.

The calculation of R_e follows the same steps, with the only difference that the starting point is the system of differential equations for the CMV model with vaccination. In the following sections we give the model equations for different vaccination scenarios and the respective disease free equilibrium.

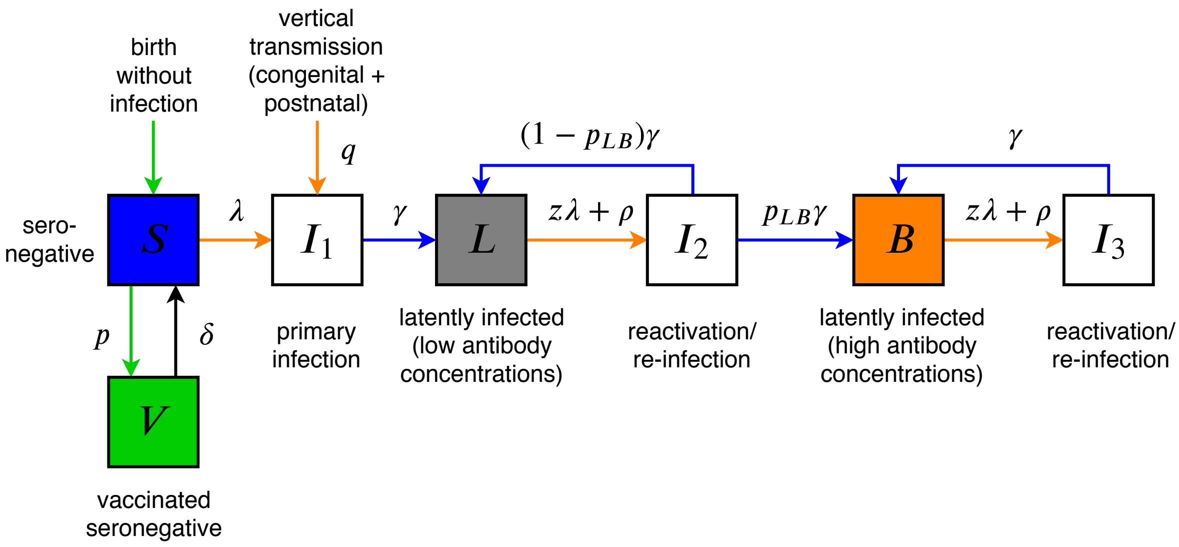

Figure S1: **Schematic of the model with universal vaccination with a vaccine preventing infection.** The vaccine is assumed to protect against primary infection in seronegative individuals. Proportion p of susceptible (S) individuals in age group k' is effectively vaccinated (V), where p is given by the product of vaccination coverage and vaccine efficacy. Vaccinated individuals lose protection at rate δ (average duration of protection, $1/\delta$), returning to the susceptible class.

6 Model with universal vaccination

We considered a suite of universal vaccination strategies with varying proportions of effectively vaccinated persons, ages at vaccination, sexes to be vaccinated, and durations of protection. We distinguished between scenarios in which the vaccine is assumed to protect only against primary infection in seronegative individuals ("prevention of infection") or against primary infection in seronegative individuals and re-infection/reactivation in seropositive individuals ("prevention of (re-)infection and reactivation"). The target population for vaccination was either infants in the first year of life, adolescent boys and girls

at the age of 10 years, adolescent girls at the age of 10 years, or women of reproductive age (15-50 years).

6.1 Prevention of infection

In the model with universal vaccination with a vaccine preventing infection, the proportion p of susceptible (seronegative, S) individuals in age group k' is effectively vaccinated (V). Henceforth, we will refer to p as the effectively vaccinated proportion, which is the product of vaccination coverage and vaccine efficacy. Vaccinated individuals lose protection at rate δ (average duration of protection, $1/\delta$), returning to the susceptible class. The special case of $\delta = 0$ describes the model with life-long protection. Schematic of this model is given in Figure S1. For different scenarios, the vaccinated age groups are $k' = 2$ (6-months-old), $k' = 4$ (10-years-old), $k' = 7$ (25-years-old).

6.1.1 6-months-old boys and girls and life-long protection

In the scenario with vaccination of infants in the first year of life, the fraction p of susceptible boys and girls (S) is vaccinated (V) at 6 months of age ($k' = 2$). In the simplest case when the vaccine-induced protection does not wane, these individuals stay protected for the rest of their lives. Let us denote $V_k^i(t)$ denote the number of vaccinated individuals of sex i in the age group k at time t. The model equations for this scenario read as follows

$$\begin{aligned}
\frac{\mathrm{d}S_1^i(t)}{\mathrm{d}t} &= \sum_{k=1}^{n} \phi_k^i \{S_k^{♀}(t) + V_k^{♀}(t) + (1-q)[I_{1,k}^{♀}(t) + L_k^{♀}(t) + I_{2,k}^{♀}(t) + B_k^{♀}(t) + I_{3,k}^{♀}(t)]\} - \\
&- \left[(1-p)m_1 + pm_1 + \lambda_1^i(t) + \mu_1^i\right] S_1^i(t), \\
\frac{\mathrm{d}V_1^i(t)}{\mathrm{d}t} &= 0, \\
\frac{\mathrm{d}S_2^i(t)}{\mathrm{d}t} &= (1-p)m_1 S_1^i(t) - \left[m_2 + \lambda_2^i(t) + \mu_2^i\right] S_2^i(t), \\
\frac{\mathrm{d}V_2^i(t)}{\mathrm{d}t} &= pm_1 S_1^i(t) - \left[m_2 + \mu_2^i\right] V_2^i(t), \\
\frac{\mathrm{d}S_k^i(t)}{\mathrm{d}t} &= m_{k-1} S_{k-1}^i(t) - \left[m_k + \lambda_k^i(t) + \mu_k^i\right] S_k^i(t), k = 3, \ldots, n \\
\frac{\mathrm{d}V_k^i(t)}{\mathrm{d}t} &= m_{k-1} V_{k-1}^i(t) - \left[m_k + \mu_k^i\right] V_k^i(t), k = 3, \ldots, n
\end{aligned}$$

$$
\begin{aligned}
\frac{\mathrm{d}I_{1,1}^i(t)}{\mathrm{d}t} &= q\sum_{k=1}^{n}\phi_k^i[I_{1,k}^{♀}(t)+L_k^{♀}(t)+I_{2,k}^{♀}(t)+B_k^{♀}(t)+I_{3,k}^{♀}(t)]+\lambda_1^i(t)S_1^i(t)-\\
&- \left[m_1+\gamma_{1,1}^i+\mu_1^i\right]I_{1,1}^i(t),\\
\frac{\mathrm{d}I_{1,k}^i(t)}{\mathrm{d}t} &= \lambda_k^i(t)S_k^i(t)+m_{k-1}I_{1,k-1}^i(t)-\left[m_k+\gamma_{1,k}^i+\mu_k^i\right]I_{1,k}^i(t),\\
\frac{\mathrm{d}L_1^i(t)}{\mathrm{d}t} &= \gamma_{1,1}^iI_{1,1}^i(t)-\left[m_1+\rho_1^i+z\lambda_1^i(t)+\mu_1^i\right]L_1^i(t)+(1-p_{LB})\gamma_{2,1}^iI_{2,1}^i(t),\\
\frac{\mathrm{d}L_k^i(t)}{\mathrm{d}t} &= \gamma_{1,k}^iI_{1,k}^i(t)+m_{k-1}L_{k-1}^i(t)-\left[m_k+\rho_k^i+z\lambda_k^i(t)+\mu_k^i\right]L_k^i(t)+\\
&+ (1-p_{LB})\gamma_{2,k}^iI_{2,k}^i(t),\\
\frac{\mathrm{d}I_{2,1}^i(t)}{\mathrm{d}t} &= \left[\rho_1^i+z\lambda_1^i(t)\right]L_1^i(t)-\left[m_1+\gamma_{2,1}^i+\mu_1^i\right]I_{2,1}^i(t),\\
\frac{\mathrm{d}I_{2,k}^i(t)}{\mathrm{d}t} &= \left[\rho_k^i+z\lambda_k^i(t)\right]L_k^i(t)+m_{k-1}I_{2,k-1}^i(t)-\left[m_k+\gamma_{2,k}^i+\mu_k^i\right]I_{2,k}^i(t),\\
\frac{\mathrm{d}B_1^i(t)}{\mathrm{d}t} &= p_{LB}\gamma_{2,1}^iI_{2,1}^i(t)-\left[m_1+\rho_1^i+z\lambda_1^i(t)+\mu_1^i\right]B_1^i(t)+\gamma_{3,1}^iI_{3,1}^i(t),\\
\frac{\mathrm{d}B_k^i(t)}{\mathrm{d}t} &= p_{LB}\gamma_{2,k}^iI_{2,k}^i(t)+m_{k-1}B_{k-1}^i(t)-\left[m_k+\rho_k^i+z\lambda_k^i(t)+\mu_k^i\right]B_k^i(t)+\\
&+ \gamma_{3,k}^iI_{3,k}^i(t),\\
\frac{\mathrm{d}I_{3,1}^i(t)}{\mathrm{d}t} &= \left[\rho_1^i+z\lambda_1^i(t)\right]B_1^i(t)-\left[m_1+\gamma_{3,1}^i+\mu_1^i\right]I_{3,1}^i(t),\\
\frac{\mathrm{d}I_{3,k}^i(t)}{\mathrm{d}t} &= \left[\rho_k^i+z\lambda_k^i(t)\right]B_k^i(t)+m_{k-1}I_{3,k-1}^i(t)-\left[m_k+\gamma_{3,k}^i+\mu_k^i\right]I_{3,k}^i(t), \qquad (100)
\end{aligned}
$$

where $k=2,\ldots,n$ in the equations for $I_{1,k}^i(t)$, $L_k^i(t)$, $I_{2,k}^i(t)$, $B_k^i(t)$ and $I_{3,k}^i(t)$.

The number of individuals of sex i in the k-th age group $[a_{k-1},a_k]$ can be written down as follows:

$$
N_k^i(t)=S_k^i(t)+I_{1,k}^i(t)+L_k^i(t)+I_{2,k}^i(t)+B_k^i(t)+I_{3,k}^i(t)+V_k^i(t), \quad k=1,\ldots,n. \quad (101)
$$

To calculate the effective reproduction number, R_e, we expressed $S_k^i(t)$ as $S_k^i(t)=N_k^i-I_{1,k}^i(t)-L_k^i(t)-I_{2,k}^i(t)-B_k^i(t)-I_{3,k}^i(t)-V_k^i(t)$ and dropped the equations for $S_k^i(t)$. The equations for the calculation of R_e are

$$\begin{aligned}
\frac{\mathrm{d}V_2^i(t)}{\mathrm{d}t} &= pm_1[N_1^i - I_{1,1}^i(t) - L_1^i(t) - I_{2,1}^i(t) - B_1^i(t) - I_{3,1}^i(t)] - m_2V_2^i(t),\\
\frac{\mathrm{d}V_k^i(t)}{\mathrm{d}t} &= m_{k-1}V_{k-1}^i(t) - m_kV_k^i(t), k = 3,\ldots,n\\
\frac{\mathrm{d}I_{1,1}^i(t)}{\mathrm{d}t} &= q\sum_{k=1}^{n}\phi_k^i[I_{1,k}^{♀}(t) + L_k^{♀}(t) + I_{2,k}^{♀}(t) + B_k^{♀}(t) + I_{3,k}^{♀}(t)] - \left[m_1 + \gamma_{1,1}^i\right] I_{1,1}^i(t) +\\
&+ \lambda_1^i(t)[N_1^i - I_{1,1}^i(t) - L_1^i(t) - I_{2,1}^i(t) - B_1^i(t) - I_{3,1}^i(t)],\\
\frac{\mathrm{d}I_{1,k}^i(t)}{\mathrm{d}t} &= \lambda_k^i(t)[N_k^i - I_{1,k}^i(t) - L_k^i(t) - I_{2,k}^i(t) - B_k^i(t) - I_{3,k}^i(t) - V_k^i(t)] +\\
&+ m_{k-1}I_{1,k-1}^i(t) - \left[m_k + \gamma_{1,k}^i\right] I_{1,k}^i(t),\\
\frac{\mathrm{d}L_1^i(t)}{\mathrm{d}t} &= \gamma_{1,1}^iI_{1,1}^i(t) - \left[m_1 + \rho_1^i + z\lambda_1^i(t)\right] L_1^i(t) + (1-p_{LB})\gamma_{2,1}^iI_{2,1}^i(t),\\
\frac{\mathrm{d}L_k^i(t)}{\mathrm{d}t} &= \gamma_{1,k}^iI_{1,k}^i(t) + m_{k-1}L_{k-1}^i(t) - \left[m_k + \rho_k^i + z\lambda_k^i(t)\right] L_k^i(t) +\\
&+ (1-p_{LB})\gamma_{2,k}^iI_{2,k}^i(t),\\
\frac{\mathrm{d}I_{2,1}^i(t)}{\mathrm{d}t} &= \left[\rho_1^i + z\lambda_1^i(t)\right] L_1^i(t) - \left[m_1 + \gamma_{2,1}^i\right] I_{2,1}^i(t),\\
\frac{\mathrm{d}I_{2,k}^i(t)}{\mathrm{d}t} &= \left[\rho_k^i + z\lambda_k^i(t)\right] L_k^i(t) + m_{k-1}I_{2,k-1}^i(t) - \left[m_k + \gamma_{2,k}^i\right] I_{2,k}^i(t),\\
\frac{\mathrm{d}B_1^i(t)}{\mathrm{d}t} &= p_{LB}\gamma_{2,1}^iI_{2,1}^i(t) - \left[m_1 + \rho_1^i + z\lambda_1^i(t)\right] B_1^i(t) + \gamma_{3,1}^iI_{3,1}^i(t),\\
\frac{\mathrm{d}B_k^i(t)}{\mathrm{d}t} &= p_{LB}\gamma_{2,k}^iI_{2,k}^i(t) + m_{k-1}B_{k-1}^i(t) - \left[m_k + \rho_k^i + z\lambda_k^i(t)\right] B_k^i(t) +\\
&+ \gamma_{3,k}^iI_{3,k}^i(t),\\
\frac{\mathrm{d}I_{3,1}^i(t)}{\mathrm{d}t} &= \left[\rho_1^i + z\lambda_1^i(t)\right] B_1^i(t) - \left[m_1 + \gamma_{3,1}^i\right] I_{3,1}^i(t),\\
\frac{\mathrm{d}I_{3,k}^i(t)}{\mathrm{d}t} &= \left[\rho_k^i + z\lambda_k^i(t)\right] B_k^i(t) + m_{k-1}I_{3,k-1}^i(t) - \left[m_k + \gamma_{3,k}^i\right] I_{3,k}^i(t), \qquad (102)
\end{aligned}$$

where $k = 2,\ldots,n$ in the equations for $I_{1,k}^i(t)$, $L_k^i(t)$, $I_{2,k}^i(t)$, $B_k^i(t)$ and $I_{3,k}^i(t)$ (all but $V_k^i(t)$).

Here

$$N_k^i = N^i\frac{(a_k - a_{k-1})}{M} \quad \text{and} \quad m_k = \frac{1}{a_k - a_{k-1}}, \quad k = 1,\ldots,n, \qquad (103)$$

and

$$\lambda_k^i(t) = \sum_{j\in\{♂,♀\}}\sum_{l=1}^{n}\tilde{c}_{kl}^{ij}\left[\beta_1I_{1,l}^j(t) + \beta_2I_{2,l}^j(t) + \beta_2I_{3,l}^j(t)\right](a_l - a_{l-1}), \quad k = 1,\ldots,n. \qquad (104)$$

The disease free equilibrium is

$$
\begin{aligned}
&\bar{S}_1^i = \frac{1}{m_1}\sum_{k=1}^{n}\phi_k^i N_k^{♀}, \quad \bar{S}_k^i = \frac{(1-p)m_1}{m_k}\bar{S}_1^i, \quad k=2,\ldots,n,\\
&\bar{V}_1^i = 0, \quad \bar{V}_k^i = \frac{pm_1}{m_k}\bar{S}_1^i, \quad k=2,\ldots,n,\\
&\bar{I}_{1,k}^i = \bar{I}_{2,k}^i = \bar{I}_{3,k}^i = \bar{L}_k^i = \bar{B}_k^i = 0, \quad k=1,\ldots,n. \qquad (105)
\end{aligned}
$$

6.1.2 6-months-old boys and girls and waning protection

If the protection wanes with rate δ, the equations for susceptible and vaccinated persons are changed:

$$
\begin{aligned}
\frac{\mathrm{d}S_2^i(t)}{\mathrm{d}t} &= (1-p)m_1 S_1^i(t) - \left[m_2 + \lambda_2^i(t) + \mu_2^i\right] S_2^i(t) + \delta V_2^i(t),\\
\frac{\mathrm{d}V_2^i(t)}{\mathrm{d}t} &= pm_1 S_1^i(t) - [\delta + m_2 + \mu_2^i]V_2^i(t),\\
\frac{\mathrm{d}S_k^i(t)}{\mathrm{d}t} &= m_{k-1}S_{k-1}^i(t) - \left[m_k + \lambda_k^i(t) + \mu_k^i\right] S_k^i(t) + \delta V_k^i(t), k=3,\ldots,n\\
\frac{\mathrm{d}V_k^i(t)}{\mathrm{d}t} &= m_{k-1}V_{k-1}^i(t) - [\delta + m_k + \mu_k^i]V_k^i(t), k=3,\ldots,n. \qquad (106)
\end{aligned}
$$

The equations for calculation of the effective reproduction number are

$$
\begin{aligned}
\frac{\mathrm{d}V_2^i(t)}{\mathrm{d}t} &= pm_1[N_1^i - I_{1,1}^i(t) - L_1^i(t) - I_{2,1}^i(t) - B_1^i(t) - I_{3,1}^i(t)] - [\delta + m_2]V_2^i(t),\\
\frac{\mathrm{d}V_k^i(t)}{\mathrm{d}t} &= m_{k-1}V_{k-1}^i(t) - [\delta + m_k]V_k^i(t), k=3,\ldots,n. \qquad (107)
\end{aligned}
$$

The disease free equilibrium is

$$
\begin{aligned}
&\bar{S}_1^i = \frac{1}{m_1}\sum_{k=1}^{n}\phi_k^i N_k^{♀},\\
&\bar{S}_2^i = \frac{m_1[\delta + (1-p)m_2]}{m_2(\delta + m_2)}\bar{S}_1^i,\\
&\bar{S}_k^i = \frac{\bar{S}_1^i}{m_k}\left(\frac{m_1[\delta + (1-p)m_2]}{(\delta + m_2)} + \delta p \sum_{l=2}^{l=k-1}\prod_{j=1}^{j=l}\frac{m_j}{\delta + m_{j+1}}\right), \quad k=3,\ldots,n,\\
&\bar{V}_1^i = 0, \quad \bar{V}_2^i = \frac{pm_1}{\delta + m_2}\bar{S}_1^i,\\
&\bar{V}_k^i = p\bar{S}_1^i \prod_{j=1}^{j=k-1}\frac{m_j}{\delta + m_{j+1}}, \quad k=3,\ldots,n,\\
&\bar{I}_{1,k}^i = \bar{I}_{2,k}^i = \bar{I}_{3,k}^i = \bar{L}_k^i = \bar{B}_k^i = 0, \quad k=1,\ldots,n. \qquad (108)
\end{aligned}
$$

6.1.3 Individuals in age class k' and waning protection

The equations for the model with vaccination of seronegative individuals in age class k' and waning protection (Figure S1) are

$$
\begin{aligned}
\frac{\mathrm{d}S_1^i(t)}{\mathrm{d}t} &= \sum_{k=1}^{n} \phi_k^i \{S_k^{♀}(t) + V_k^{♀}(t) + (1-q)[I_{1,k}^{♀}(t) + L_k^{♀}(t) + I_{2,k}^{♀}(t) + B_k^{♀}(t) + I_{3,k}^{♀}(t)]\} - \\
&- \left[m_1 + \lambda_1^i(t) + \mu_1^i\right] S_1^i(t), \\
\frac{\mathrm{d}S_k^i(t)}{\mathrm{d}t} &= m_{k-1}S_{k-1}^i(t) - \left[m_k + \lambda_k^i(t) + \mu_k^i\right] S_k^i(t), \quad k = 2, \ldots, (k'-1) \\
\frac{\mathrm{d}V_k^i(t)}{\mathrm{d}t} &= 0, \quad k = 1, \ldots, (k'-1) \\
\frac{\mathrm{d}S_{k'}^i(t)}{\mathrm{d}t} &= (1-p)m_{k'-1}S_{k'-1}^i(t) - \left[m_{k'} + \lambda_{k'}^i(t) + \mu_{k'}^i\right] S_{k'}^i(t) + \delta V_{k'}^i(t), \\
\frac{\mathrm{d}V_{k'}^i(t)}{\mathrm{d}t} &= pm_{k'-1}S_{k'-1}^i(t) - \left[\delta + m_{k'} + \mu_{k'}^i\right] V_{k'}^i(t), \\
\frac{\mathrm{d}S_r^i(t)}{\mathrm{d}t} &= m_{r-1}S_{r-1}^i(t) - \left[m_r + \lambda_r^i(t) + \mu_r^i\right] S_r^i(t) + \delta V_r^i(t), r = (k'+1), (k'+2), \ldots, n \\
\frac{\mathrm{d}V_r^i(t)}{\mathrm{d}t} &= m_{r-1}V_{r-1}^i(t) - \left[\delta + m_r + \mu_r^i\right] V_r^i(t), \quad r = (k'+1), (k'+2), \ldots, n \\
\frac{\mathrm{d}I_{1,1}^i(t)}{\mathrm{d}t} &= q\sum_{k=1}^{n} \phi_k^i [I_{1,k}^{♀}(t) + L_k^{♀}(t) + I_{2,k}^{♀}(t) + B_k^{♀}(t) + I_{3,k}^{♀}(t)] + \lambda_1^i(t)S_1^i(t) - \\
&- \left[m_1 + \gamma_{1,1}^i + \mu_1^i\right] I_{1,1}^i(t), \\
\frac{\mathrm{d}I_{1,k}^i(t)}{\mathrm{d}t} &= \lambda_k^i(t)S_k^i(t) + m_{k-1}I_{1,k-1}^i(t) - \left[m_k + \gamma_{1,k}^i + \mu_k^i\right] I_{1,k}^i(t), \\
\frac{\mathrm{d}L_1^i(t)}{\mathrm{d}t} &= \gamma_{1,1}^i I_{1,1}^i(t) - \left[m_1 + \rho_1^i + z\lambda_1^i(t) + \mu_1^i\right] L_1^i(t) + (1-p_{LB})\gamma_{2,1}^i I_{2,1}^i(t), \\
\frac{\mathrm{d}L_k^i(t)}{\mathrm{d}t} &= \gamma_{1,k}^i I_{1,k}^i(t) + m_{k-1}L_{k-1}^i(t) - \left[m_k + \rho_k^i + z\lambda_k^i(t) + \mu_k^i\right] L_k^i(t) + \\
&+ (1-p_{LB})\gamma_{2,k}^i I_{2,k}^i(t), \\
\frac{\mathrm{d}I_{2,1}^i(t)}{\mathrm{d}t} &= \left[\rho_1^i + z\lambda_1^i(t)\right] L_1^i(t) - \left[m_1 + \gamma_{2,1}^i + \mu_1^i\right] I_{2,1}^i(t), \\
\frac{\mathrm{d}I_{2,k}^i(t)}{\mathrm{d}t} &= \left[\rho_k^i + z\lambda_k^i(t)\right] L_k^i(t) + m_{k-1}I_{2,k-1}^i(t) - \left[m_k + \gamma_{2,k}^i + \mu_k^i\right] I_{2,k}^i(t), \\
\frac{\mathrm{d}B_1^i(t)}{\mathrm{d}t} &= p_{LB}\gamma_{2,1}^i I_{2,1}^i(t) - \left[m_1 + \rho_1^i + z\lambda_1^i(t) + \mu_1^i\right] B_1^i(t) + \gamma_{3,1}^i I_{3,1}^i(t), \\
\frac{\mathrm{d}B_k^i(t)}{\mathrm{d}t} &= p_{LB}\gamma_{2,k}^i I_{2,k}^i(t) + m_{k-1}B_{k-1}^i(t) - \left[m_k + \rho_k^i + z\lambda_k^i(t) + \mu_k^i\right] B_k^i(t) + \\
&+ \gamma_{3,k}^i I_{3,k}^i(t),
\end{aligned}
$$

$$\frac{\mathrm{d}I^i_{3,1}(t)}{\mathrm{d}t} = \left[\rho^i_1 + z\lambda^i_1(t)\right] B^i_1(t) - \left[m_1 + \gamma^i_{3,1} + \mu^i_1\right] I^i_{3,1}(t),$$
$$\frac{\mathrm{d}I^i_{3,k}(t)}{\mathrm{d}t} = \left[\rho^i_k + z\lambda^i_k(t)\right] B^i_k(t) + m_{k-1}I^i_{3,k-1}(t) - \left[m_k + \gamma^i_{3,k} + \mu^i_k\right] I^i_{3,k}(t), \quad (109)$$

where $k = 2, \ldots, n$ in the equations for $I^i_{1,k}(t)$, $L^i_k(t)$, $I^i_{2,k}(t)$, $B^i_k(t)$ and $I^i_{3,k}(t)$.

The model equations for the calculation of R_e (without the equations for susceptibles):

$$\begin{aligned}
\frac{\mathrm{d}V^i_{k'}(t)}{\mathrm{d}t} &= pm_{k'-1}\left[N^i_{k'-1} - I^i_{1,k'-1}(t) - L^i_{k'-1}(t) - I^i_{2,k'-1}(t) - B^i_{k'-1}(t) - I^i_{3,k'-1}(t)\right] - \\
&- \left[\delta + m_{k'}\right] V^i_{k'}(t), \\
\frac{\mathrm{d}V^i_r(t)}{\mathrm{d}t} &= m_{r-1}V^i_{r-1}(t) - \left[\delta + m_r\right] V^i_r(t), \quad r = (k'+1), (k'+2), \ldots, n \\
\frac{\mathrm{d}I^i_{1,1}(t)}{\mathrm{d}t} &= q\sum_{k=1}^{n}\phi^i_k[I^{♀}_{1,k}(t) + L^{♀}_k(t) + I^{♀}_{2,k}(t) + B^{♀}_k(t) + I^{♀}_{3,k}(t)] - \left[m_1 + \gamma^i_{1,1}\right] I^i_{1,1}(t) + \\
&+ \lambda^i_1(t)[N^i_1 - I^i_{1,1}(t) - L^i_1(t) - I^i_{2,1}(t) - B^i_1(t) - I^i_{3,1}(t)], \\
\frac{\mathrm{d}I^i_{1,k}(t)}{\mathrm{d}t} &= \lambda^i_k(t)[N^i_k - I^i_{1,k}(t) - L^i_k(t) - I^i_{2,k}(t) - B^i_k(t) - I^i_{3,k}(t)] + m_{k-1}I^i_{1,k-1}(t) - \\
&- \left[m_k + \gamma^i_{1,k}\right] I^i_{1,k}(t), \quad k = 2, 3, \ldots, (k'-1) \\
\frac{\mathrm{d}I^i_{1,r}(t)}{\mathrm{d}t} &= \lambda^i_r(t)[N^i_r - I^i_{1,r}(t) - L^i_r(t) - I^i_{2,r}(t) - B^i_r(t) - I^i_{3,r}(t) - V^i_r(t)] + m_{r-1}I^i_{1,r-1}(t) - \\
&- \left[m_r + \gamma^i_{1,r}\right] I^i_{1,r}(t), \quad r = k', (k'+1), \ldots, n \\
\frac{\mathrm{d}L^i_1(t)}{\mathrm{d}t} &= \gamma^i_{1,1}I^i_{1,1}(t) - \left[m_1 + \rho^i_1 + z\lambda^i_1(t)\right] L^i_1(t) + (1 - p_{LB})\gamma^i_{2,1}I^i_{2,1}(t), \\
\frac{\mathrm{d}L^i_k(t)}{\mathrm{d}t} &= \gamma^i_{1,k}I^i_{1,k}(t) + m_{k-1}L^i_{k-1}(t) - \left[m_k + \rho^i_k + z\lambda^i_k(t)\right] L^i_k(t) + \\
&+ (1 - p_{LB})\gamma^i_{2,k}I^i_{2,k}(t), \\
\frac{\mathrm{d}I^i_{2,1}(t)}{\mathrm{d}t} &= \left[\rho^i_1 + z\lambda^i_1(t)\right] L^i_1(t) - \left[m_1 + \gamma^i_{2,1}\right] I^i_{2,1}(t), \\
\frac{\mathrm{d}I^i_{2,k}(t)}{\mathrm{d}t} &= \left[\rho^i_k + z\lambda^i_k(t)\right] L^i_k(t) + m_{k-1}I^i_{2,k-1}(t) - \left[m_k + \gamma^i_{2,k}\right] I^i_{2,k}(t), \\
\frac{\mathrm{d}B^i_1(t)}{\mathrm{d}t} &= p_{LB}\gamma^i_{2,1}I^i_{2,1}(t) - \left[m_1 + \rho^i_1 + z\lambda^i_1(t)\right] B^i_1(t) + \gamma^i_{3,1}I^i_{3,1}(t), \\
\frac{\mathrm{d}B^i_k(t)}{\mathrm{d}t} &= p_{LB}\gamma^i_{2,k}I^i_{2,k}(t) + m_{k-1}B^i_{k-1}(t) - \left[m_k + \rho^i_k + z\lambda^i_k(t)\right] B^i_k(t) + \\
&+ \gamma^i_{3,k}I^i_{3,k}(t), \\
\frac{\mathrm{d}I^i_{3,1}(t)}{\mathrm{d}t} &= \left[\rho^i_1 + z\lambda^i_1(t)\right] B^i_1(t) - \left[m_1 + \gamma^i_{3,1}\right] I^i_{3,1}(t), \\
\frac{\mathrm{d}I^i_{3,k}(t)}{\mathrm{d}t} &= \left[\rho^i_k + z\lambda^i_k(t)\right] B^i_k(t) + m_{k-1}I^i_{3,k-1}(t) - \left[m_k + \gamma^i_{3,k}\right] I^i_{3,k}(t), \quad (110)
\end{aligned}$$

where $k = 2, \ldots, n$ in the equations for $L^i_k(t)$, $I^i_{2,k}(t)$, $B^i_k(t)$ and $I^i_{3,k}(t)$.

The disease free equilibrium for the calculation of R_e is

$$\begin{aligned}
\bar{S}_1^i &= \frac{1}{m_1}\sum_{k=1}^{n}\phi_k^i N_k^{\female},\\
\bar{S}_k^i &= \frac{m_1}{m_k}\bar{S}_1^i, \quad k = 2,\ldots,(k'-1)\\
\bar{V}_k^i &= 0, \quad k = 1,2,\ldots,(k'-1)\\
\bar{S}_{k'}^i &= \frac{m_1[\delta+(1-p)m_{k'}]}{m_{k'}(\delta+m_{k'})}\bar{S}_1^i,\\
\bar{V}_{k'}^i &= \frac{pm_1}{\delta+m_{k'}}\bar{S}_1^i,\\
\bar{V}_r^i &= \frac{m_{r-1}}{\delta+m_r}\bar{V}_{r-1}^i, \quad r = (k'+1),(k'+2),\ldots,n\\
\bar{S}_r^i &= \frac{m_{r-1}}{m_r}\left(\bar{S}_{r-1}^i+\frac{\delta}{\delta+m_r}\bar{V}_{r-1}^i\right), \quad r = (k'+1),(k'+2),\ldots,n\\
\bar{I}_{1,k}^i &= \bar{I}_{2,k}^i = \bar{I}_{3,k}^i = \bar{L}_k^i = \bar{B}_k^i = 0, \quad k = 1,\ldots,n.
\end{aligned} \tag{111}$$

6.2 Prevention of (re-)infection and reactivation

In the model with universal vaccination with a vaccine preventing (re-)infection and reactivation, the proportion p of seronegative (S) and seropositive individuals (L, B, I_1, I_2, and I_3) individuals in age group k' is effectively vaccinated. The seronegative and seropositive vaccinated individuals transit to class V_1 and V_2, respectively. As before, p is the effectively vaccinated proportion, which is the product of vaccination coverage and vaccine efficacy. Vaccinated individuals (V_1 and V_2) lose protection at rate δ (average duration of protection, $1/\delta$), returning from class V_1 to the susceptible class (S) and from class V_2 to the latent class with low antibody concentrations (L). Schematic of this model is given in Figure S2. For different scenarios, the vaccinated age groups are $k' = 2$ (6-months-old), $k' = 4$ (10-years-old), $k' = 7$ (25-years-old).

6.2.1 Individuals in age class k' and waning protection

The model equations are

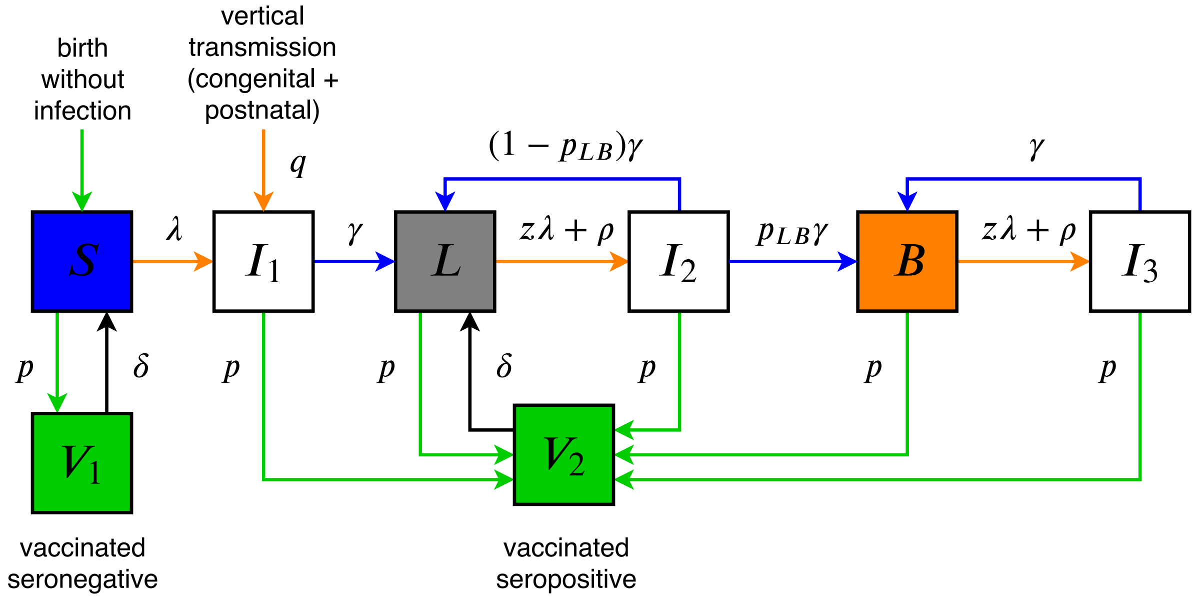

Figure S2: **Schematic of the model with universal vaccination with a vaccine preventing (re-)infection and reactivation.** The vaccine is assumed to protect against primary infection in seronegative individuals and re-infection/reactivation in seropositive individuals. In this vaccination scenario, the proportion p of susceptible individuals (S) in age group k' is effectively vaccinated (V_1). The proportion p of all seropositive persons (L, B, I_1, I_2, and I_3) in age group k' is vaccinated (V_2) as well. After the protection is lost (average duration: $1/\delta$), V_1 and V_2 individuals return to the S and L class, respectively.

$$
\begin{aligned}
\frac{\mathrm{d}S_1^i(t)}{\mathrm{d}t} &= \sum_{k=1}^{n} \phi_k^i \{S_k^{♀}(t) + V_{1,k}^{♀}(t) + V_{2,k}^{♀}(t) + (1-q)[I_{1,k}^{♀}(t) + L_k^{♀}(t) + I_{2,k}^{♀}(t) + B_k^{♀}(t) + \\
&+ I_{3,k}^{♀}(t)]\} - \left[m_1 + \lambda_1^i(t) + \mu_1^i\right] S_1^i(t), \\
\frac{\mathrm{d}S_k^i(t)}{\mathrm{d}t} &= m_{k-1} S_{k-1}^i(t) - \left[m_k + \lambda_k^i(t) + \mu_k^i\right] S_k^i(t), \quad k = 2, \ldots, (k'-1) \\
\frac{\mathrm{d}V_{1,k}^i(t)}{\mathrm{d}t} &= 0, \quad k = 1, \ldots, (k'-1) \\
\frac{\mathrm{d}S_{k'}^i(t)}{\mathrm{d}t} &= (1-p) m_{k'-1} S_{k'-1}^i(t) - \left[m_{k'} + \lambda_{k'}^i(t) + \mu_{k'}^i\right] S_{k'}^i(t) + \delta V_{1,k'}^i(t), \\
\frac{\mathrm{d}V_{1,k'}^i(t)}{\mathrm{d}t} &= p m_{k'-1} S_{k'-1}^i(t) - \left[\delta + m_{k'} + \mu_{k'}^i\right] V_{1,k'}^i(t), \\
\frac{\mathrm{d}S_r^i(t)}{\mathrm{d}t} &= m_{r-1} S_{r-1}^i(t) - \left[m_r + \lambda_r^i(t) + \mu_r^i\right] S_r^i(t) + \delta V_{1,r}^i(t), r = (k'+1), (k'+2), \ldots, n \\
\frac{\mathrm{d}V_{1,r}^i(t)}{\mathrm{d}t} &= m_{r-1} V_{1,r-1}^i(t) - \left[\delta + m_r + \mu_r^i\right] V_{1,r}^i(t), \quad r = (k'+1), (k'+2), \ldots, n \\
\frac{\mathrm{d}V_{2,k}^i(t)}{\mathrm{d}t} &= 0, \quad k = 1, \ldots, (k'-1) \\
\frac{\mathrm{d}V_{2,k'}^i(t)}{\mathrm{d}t} &= p m_{k'-1} \left[I_{1,k'-1}^i(t) + L_{k'-1}^i(t) + I_{2,k'-1}^i(t) + B_{k'-1}^i(t) + I_{3,k'-1}^i(t)\right] - \\
&- \left[\delta + m_{k'} + \mu_{k'}^i\right] V_{2,k'}^i(t), \\
\frac{\mathrm{d}V_{2,r}^i(t)}{\mathrm{d}t} &= m_{r-1} V_{2,r-1}^i(t) - \left[\delta + m_r + \mu_r^i\right] V_{2,r}^i(t), \quad r = (k'+1), (k'+2), \ldots, n \\
\frac{\mathrm{d}I_{1,1}^i(t)}{\mathrm{d}t} &= q \sum_{k=1}^{n} \phi_k^i [I_{1,k}^{♀}(t) + L_k^{♀}(t) + I_{2,k}^{♀}(t) + B_k^{♀}(t) + I_{3,k}^{♀}(t)] + \lambda_1^i(t) S_1^i(t) - \\
&- \left[m_1 + \gamma_{1,1}^i + \mu_1^i\right] I_{1,1}^i(t), \\
\frac{\mathrm{d}I_{1,k}^i(t)}{\mathrm{d}t} &= \lambda_k^i(t) S_k^i(t) + (1 - p\chi_{k,k'}) m_{k-1} I_{1,k-1}^i(t) - \left[m_k + \gamma_{1,k}^i + \mu_k^i\right] I_{1,k}^i(t), \\
\frac{\mathrm{d}L_1^i(t)}{\mathrm{d}t} &= \gamma_{1,1}^i I_{1,1}^i(t) - \left[m_1 + \rho_1^i + z\lambda_1^i(t) + \mu_1^i\right] L_1^i(t) + (1 - p_{LB}) \gamma_{2,1}^i I_{2,1}^i(t), \\
\frac{\mathrm{d}L_k^i(t)}{\mathrm{d}t} &= \gamma_{1,k}^i I_{1,k}^i(t) + (1 - p\chi_{k,k'}) m_{k-1} L_{k-1}^i(t) - \left[m_k + \rho_k^i + z\lambda_k^i(t) + \mu_k^i\right] L_k^i(t) + \\
&+ (1 - p_{LB}) \gamma_{2,k}^i I_{2,k}^i(t) + \delta V_{2,k}^i(t), \\
\frac{\mathrm{d}I_{2,1}^i(t)}{\mathrm{d}t} &= \left[\rho_1^i + z\lambda_1^i(t)\right] L_1^i(t) - \left[m_1 + \gamma_{2,1}^i + \mu_1^i\right] I_{2,1}^i(t), \\
\frac{\mathrm{d}I_{2,k}^i(t)}{\mathrm{d}t} &= \left[\rho_k^i + z\lambda_k^i(t)\right] L_k^i(t) + (1 - p\chi_{k,k'}) m_{k-1} I_{2,k-1}^i(t) - \left[m_k + \gamma_{2,k}^i + \mu_k^i\right] I_{2,k}^i(t),
\end{aligned}
$$

$$
\begin{aligned}
\frac{\mathrm{d}B_1^i(t)}{\mathrm{d}t} &= p_{LB}\gamma_{2,1}^i I_{2,1}^i(t) - \left[m_1 + \rho_1^i + z\lambda_1^i(t) + \mu_1^i\right] B_1^i(t) + \gamma_{3,1}^i I_{3,1}^i(t),\\
\frac{\mathrm{d}B_k^i(t)}{\mathrm{d}t} &= p_{LB}\gamma_{2,k}^i I_{2,k}^i(t) + (1 - p\chi_{k,k'})m_{k-1}B_{k-1}^i(t) - \left[m_k + \rho_k^i + z\lambda_k^i(t) + \mu_k^i\right] B_k^i(t) +\\
&+ \gamma_{3,k}^i I_{3,k}^i(t),\\
\frac{\mathrm{d}I_{3,1}^i(t)}{\mathrm{d}t} &= \left[\rho_1^i + z\lambda_1^i(t)\right] B_1^i(t) - \left[m_1 + \gamma_{3,1}^i + \mu_1^i\right] I_{3,1}^i(t),\\
\frac{\mathrm{d}I_{3,k}^i(t)}{\mathrm{d}t} &= \left[\rho_k^i + z\lambda_k^i(t)\right] B_k^i(t) + (1 - p\chi_{k,k'})m_{k-1}I_{3,k-1}^i(t) - \left[m_k + \gamma_{3,k}^i + \mu_k^i\right] I_{3,k}^i(t) \quad (112)
\end{aligned}
$$

where $k = 2, \ldots, n$ in the equations for $I_{1,k}^i(t)$, $L_k^i(t)$, $I_{2,k}^i(t)$, $B_k^i(t)$ and $I_{3,k}^i(t)$.

The equations for the calculation of R_e (without the equations for susceptibles) are

$$
\begin{aligned}
\frac{\mathrm{d}V_{1,k'}^i(t)}{\mathrm{d}t} &= pm_{k'-1}\left[N_{k'-1}^i - I_{1,k'-1}^i(t) - L_{k'-1}^i(t) - I_{2,k'-1}^i(t) - B_{k'-1}^i(t) - I_{3,k'-1}^i(t)\right] -\\
&- \left[\delta + m_{k'}\right] V_{1,k'}^i(t),\\
\frac{\mathrm{d}V_{1,r}^i(t)}{\mathrm{d}t} &= m_{r-1}V_{1,r-1}^i(t) - \left[\delta + m_r\right] V_{1,r}^i(t), \quad r = (k'+1), (k'+2), \ldots, n\\
\frac{\mathrm{d}V_{2,k'}^i(t)}{\mathrm{d}t} &= pm_{k'-1}\left[I_{1,k'-1}^i(t) + L_{k'-1}^i(t) + I_{2,k'-1}^i(t) + B_{k'-1}^i(t) + I_{3,k'-1}^i(t)\right] -\\
&- \left[\delta + m_{k'}\right] V_{2,k'}^i(t),\\
\frac{\mathrm{d}V_{2,r}^i(t)}{\mathrm{d}t} &= m_{r-1}V_{2,r-1}^i(t) - \left[\delta + m_r\right] V_{2,r}^i(t), \quad r = (k'+1), (k'+2), \ldots, n\\
\frac{\mathrm{d}I_{1,1}^i(t)}{\mathrm{d}t} &= q\sum_{k=1}^{n} \phi_k^i[I_{1,k}^{♀}(t) + L_k^{♀}(t) + I_{2,k}^{♀}(t) + B_k^{♀}(t) + I_{3,k}^{♀}(t)] - \left[m_1 + \gamma_{1,1}^i\right] I_{1,1}^i(t) +\\
&+ \lambda_1^i(t)[N_1^i - I_{1,1}^i(t) - L_1^i(t) - I_{2,1}^i(t) - B_1^i(t) - I_{3,1}^i(t)],\\
\frac{\mathrm{d}I_{1,k}^i(t)}{\mathrm{d}t} &= \lambda_k^i(t)[N_k^i - I_{1,k}^i(t) - L_k^i(t) - I_{2,k}^i(t) - B_k^i(t) - I_{3,k}^i(t)] + m_{k-1}I_{1,k-1}^i(t) -\\
&- \left[m_k + \gamma_{1,k}^i\right] I_{1,k}^i(t), \quad k = 2, 3, \ldots, (k'-1)\\
\frac{\mathrm{d}I_{1,r}^i(t)}{\mathrm{d}t} &= \lambda_r^i(t)[N_r^i - I_{1,r}^i(t) - L_r^i(t) - I_{2,r}^i(t) - B_r^i(t) - I_{3,r}^i(t) - V_{1,r}^i(t) - V_{2,r}^i(t)] +\\
&+ (1 - p\chi_{r,k'})m_{r-1}I_{1,r-1}^i(t) - \left[m_r + \gamma_{1,r}^i\right] I_{1,r}^i(t), \quad r = k', (k'+1), \ldots, n\\
\frac{\mathrm{d}L_1^i(t)}{\mathrm{d}t} &= \gamma_{1,1}^i I_{1,1}^i(t) - \left[m_1 + \rho_1^i + z\lambda_1^i(t)\right] L_1^i(t) + (1 - p_{LB})\gamma_{2,1}^i I_{2,1}^i(t),\\
\frac{\mathrm{d}L_k^i(t)}{\mathrm{d}t} &= \gamma_{1,k}^i I_{1,k}^i(t) + (1 - p\chi_{k,k'})m_{k-1}L_{k-1}^i(t) - \left[m_k + \rho_k^i + z\lambda_k^i(t)\right] L_k^i(t) +\\
&+ (1 - p_{LB})\gamma_{2,k}^i I_{2,k}^i(t) + \delta V_{2,k}^i(t),
\end{aligned}
$$

$$
\begin{aligned}
\frac{\mathrm{d}I^i_{2,1}(t)}{\mathrm{d}t} &= \left[\rho^i_1 + z\lambda^i_1(t)\right] L^i_1(t) - \left[m_1 + \gamma^i_{2,1}\right] I^i_{2,1}(t), \\
\frac{\mathrm{d}I^i_{2,k}(t)}{\mathrm{d}t} &= \left[\rho^i_k + z\lambda^i_k(t)\right] L^i_k(t) + (1 - p\chi_{k,k'})m_{k-1}I^i_{2,k-1}(t) - \left[m_k + \gamma^i_{2,k}\right] I^i_{2,k}(t), \\
\frac{\mathrm{d}B^i_1(t)}{\mathrm{d}t} &= p_{LB}\gamma^i_{2,1}I^i_{2,1}(t) - \left[m_1 + \rho^i_1 + z\lambda^i_1(t)\right] B^i_1(t) + \gamma^i_{3,1}I^i_{3,1}(t), \\
\frac{\mathrm{d}B^i_k(t)}{\mathrm{d}t} &= p_{LB}\gamma^i_{2,k}I^i_{2,k}(t) + (1 - p\chi_{k,k'})m_{k-1}B^i_{k-1}(t) - \left[m_k + \rho^i_k + z\lambda^i_k(t)\right] B^i_k(t) + \\
&+ \gamma^i_{3,k}I^i_{3,k}(t), \\
\frac{\mathrm{d}I^i_{3,1}(t)}{\mathrm{d}t} &= \left[\rho^i_1 + z\lambda^i_1(t)\right] B^i_1(t) - \left[m_1 + \gamma^i_{3,1}\right] I^i_{3,1}(t), \\
\frac{\mathrm{d}I^i_{3,k}(t)}{\mathrm{d}t} &= \left[\rho^i_k + z\lambda^i_k(t)\right] B^i_k(t) + (1 - p\chi_{k,k'})m_{k-1}I^i_{3,k-1}(t) - \left[m_k + \gamma^i_{3,k}\right] I^i_{3,k}(t), \quad (113)
\end{aligned}
$$

where $k = 2, \ldots, n$, $2 \leq k' \leq n$ in the equations for $L^i_k(t)$, $I^i_{2,k}(t)$, $B^i_k(t)$ and $I^i_{3,k}(t)$. $\chi_{k,k'}$ is the kronecker symbol: $\chi_{k,k'} = 1, k = k'$ and $\chi_{k,k'} = 0$ otherwise.

The disease free steady state for the calculation of R_e is

$$
\begin{aligned}
&\bar{S}^i_1 = \frac{1}{m_1}\sum_{k=1}^{n}\phi^i_k N^{♀}_k, \\
&\bar{S}^i_k = \frac{m_1}{m_k}\bar{S}^i_1, \quad k = 2, \ldots, (k'-1) \\
&\bar{V}^i_{1,k} = 0, \quad k = 1, 2, \ldots, (k'-1) \\
&\bar{S}^i_{k'} = \frac{1}{m_{k'}}\left[(1-p)m_1\bar{S}^i_1 + \frac{\delta p m_{k'-1}}{\delta + m_{k'}}N^i_{k'-1}\right], \\
&\bar{V}^i_{1,k'} = \frac{p m_{k'-1}}{\delta + m_{k'}}N^i_{k'-1}, \\
&\bar{V}^i_{1,r} = \frac{m_{r-1}}{\delta + m_r}\bar{V}^i_{1,r-1}, \quad r = (k'+1), (k'+2), \ldots, n \\
&\bar{S}^i_r = \frac{m_{r-1}}{m_r}\left(\bar{S}^i_{r-1} + \frac{\delta}{\delta + m_r}\bar{V}^i_{1,r-1}\right), \quad r = (k'+1), (k'+2), \ldots, n \\
&\bar{V}^i_{2,k} = \bar{I}^i_{1,k} = \bar{I}^i_{2,k} = \bar{I}^i_{3,k} = \bar{L}^i_k = \bar{B}^i_k = 0, \quad k = 1, \ldots, n. \qquad (114)
\end{aligned}
$$

7 Model outcomes

We evaluated the impact of all interventions on the percent reduction in the incidence of cCMV, primary infections and re-infections/reactivations after a time frame of 20 years.

The incidence of primary infections at time t was computed as

$$
\sum_{k=1}^{n}\sum_{i\in\{♂,♀\}}\lambda^i_k(t)S^i_k(t). \qquad (115)
$$

The incidence of re-infections/reactivations at time t was computed as

$$\sum_{k=1}^{n} \sum_{i \in \{♂,♀\}} [\rho_k^i + z\lambda_k^i(t)][L_k^i(t) + B_k^i(t)]. \tag{116}$$

The incidence of congenital infections at time t was computed as

$$\gamma q_c \sum_{k=1}^{n} \sum_{i \in \{♂,♀\}} \phi_k^i [I_{1,k}^{♀}(t) + I_{2,k}^{♀}(t) + I_{3,k}^{♀}(t)], \tag{117}$$

where $1/\gamma$ is the duration of acute infection.

8 Parameter summary

The description of the model parameters is given in Table S1.

Notation	Unit	Description	Value/Source
μ_k^i	1/year	death rate of persons of sex i in age group k	$\mu_k^i = 0$ (Type I mortality)
M	years	maximum attainable age	80 years (max. age in serological data)
N		total population size	17 million, Statistics Netherlands
ϕ_k^i	1/year	women's fertility rate	Statistics Netherlands
$\tilde{c}_{kl}^{ij}$	1/day	number of contacts per day between one individual of sex i in age group k and one individual of sex j in age group l	[41]
$q \in [0,1]$		probability of vertical transmission	estimated
$q_c \in [0,1]$		probability of congenital infection	estimated
λ_k^i	1/year	force of infection for persons of sex i in age group k	estimated
ρ_k^i	1/year	reactivation rate for persons of sex i in age group k	estimated
$z \in [0,1]$		reduction in susceptibility to re-infection in latently infected persons compared to seronegative persons	estimated
$p_{LB} \in [0,1]$		probability of progression from low to high antibody concentrations	estimated
$1/\gamma_{1,k}^i = 1/\gamma$	days		
$1/\gamma_{2,k}^i = 1/\gamma$	days	duration of acute infection	14 days, sensitivity analyses
$1/\gamma_{3,k}^i = 1/\gamma$	days		
β_1	1/year	infectivity of primary infection	estimated
β_2	1/year	infectivity of re-infection/reactivation	estimated
p		effectively vaccinated proportion (vaccine efficacy × vaccination coverage)	0—100%, sensitivity analyses
$1/\delta$	years	duration of protection	2.5—80 years, sensitivity analyses

Table S1: **Summary of the model parameters**

9 Parameter inference

Parameters estimates of the transmission model were obtained using the cross-sectional serological data providing information on the infection status of the Dutch population in 2006/2007, and a retrospective cohort study carried out in 2008 providing information on the birth prevalence of cCMV (i.e. the fraction of infants infected during

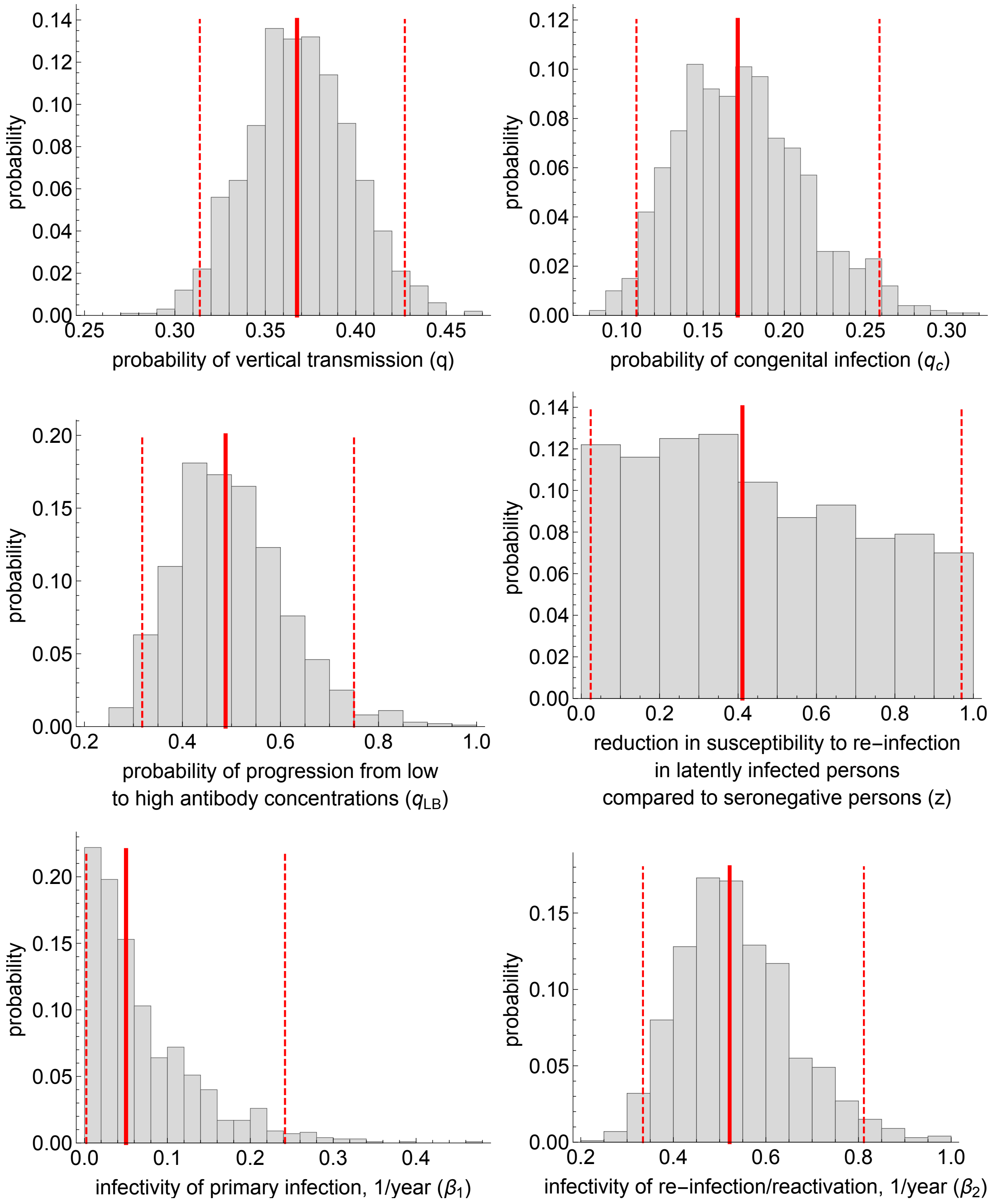

Figure S3: **Histogram of the estimated parameter values.** The solid lines are the median estimates. The dashed lines correspond to 95% credible intervals obtained from 1000 parameter samples from the posterior distribution.

pregnancy) [33,34]. Parameters were broadly estimated using methods developed earlier [41]. Briefly, assuming an endemic equilibrium and the short disease approximation, estimates were obtained using a Bayesian framework. In comparison with our earlier analyses, we extended the model by (i) allowing for multiple reactivation and re-infection events occurring over a person's life (Figure 1 in the main text), (ii) using cubic B-splines for more flexible estimation of the age-dependent reactivation rates, and (iii) including the birth cohort data to enable estimation of the probability of cCMV. For horizontal transmission we used an age- and sex-specific contact matrix with 17 age classes. The model is fitted to the data using Hamiltonian Monte Carlo method as implemented in Stan (`https://mc-stan.org`). Data, model code, and Rmarkdown file are available at `https://github.com/mvboven/cmv-vaccination`. The Rmarkdown file with prior specifications and parameter settings is attached at the end of the Supplementary Material.

Figure S3 shows an overview of all parameter estimates which are not given in the main text.

10 Results for the scenario with high reactivation rates

Estimates of the reactivation rates, infectivity of re-infection and reactivation, and probability to move from the L to B class depend sensitively on the assumed prior distributions of the reactivation rates. In the main analyses, we took prior distributions such that a priori reactivation rates range from 0.005 to 0.11 per year (95% range), which seemed reasonable given what we had found earlier [41].However, with our more flexible model which allows for multiple reactivation and re-infection events in the infected classes (L and B), the reactivation rate estimates are found to depend sensitively on the prior distributions (Figure S4). For instance, with broader and higher prior distributions for these rates, the posterior distributions of the reactivation rates are also (markedly) higher, while the infectivity and probability to move from the L to the B class are (markedly) lower (Figure S5). Fortunately, due to the neutralizing effects of the parameter shifts, the impact of vaccination, in particular the estimated reductions in cCMV, primary infections, re-infections, and reactivations are quantitatively very similar to the main scenario and sensitivity analyses (cf. Table 1 in the main text and Table S2). Hence, while considerable uncertainty surrounds the actual magnitude of the reactivation rates and associated parameters, the impact on the effectiveness of vaccination is small.

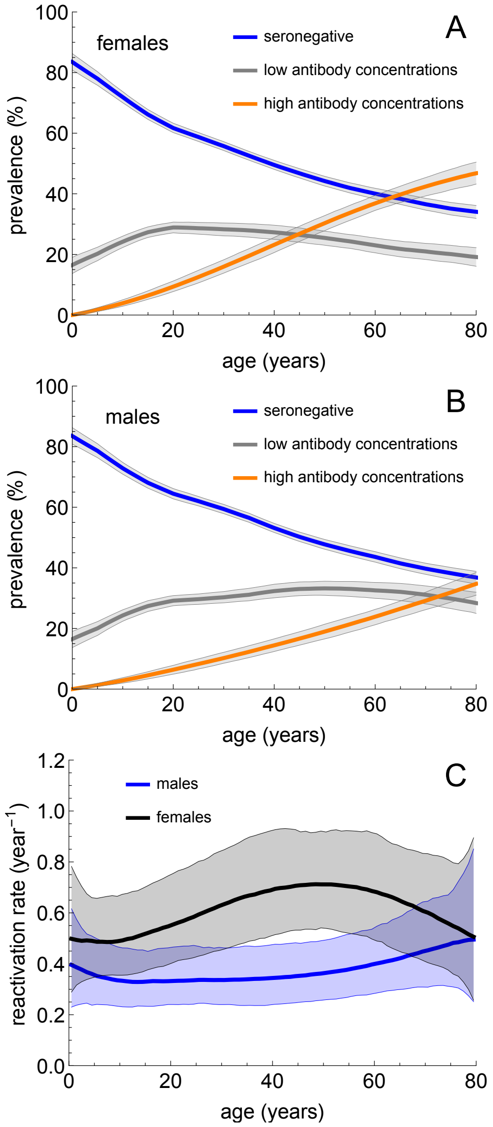

Figure S4: **Overview of the estimation results in the scenario with high reactivation rates.** (A) and (B) show the age-specific prevalence of seronegative, seropositive with low antibody concentrations and seropositive with high antibody concentrations in females (A) and males (B), respectively. (C) shows the age-specific reactivation rates per year for females (black) and males (blue), respectively. The solid lines are the median estimates, and the shaded regions correspond to 95% credible intervals obtained from 1000 parameter samples from the posterior distribution. Note that the seroprevalence is estimated with high precision, and that credible intervals for the reactivation rates are quite broad.

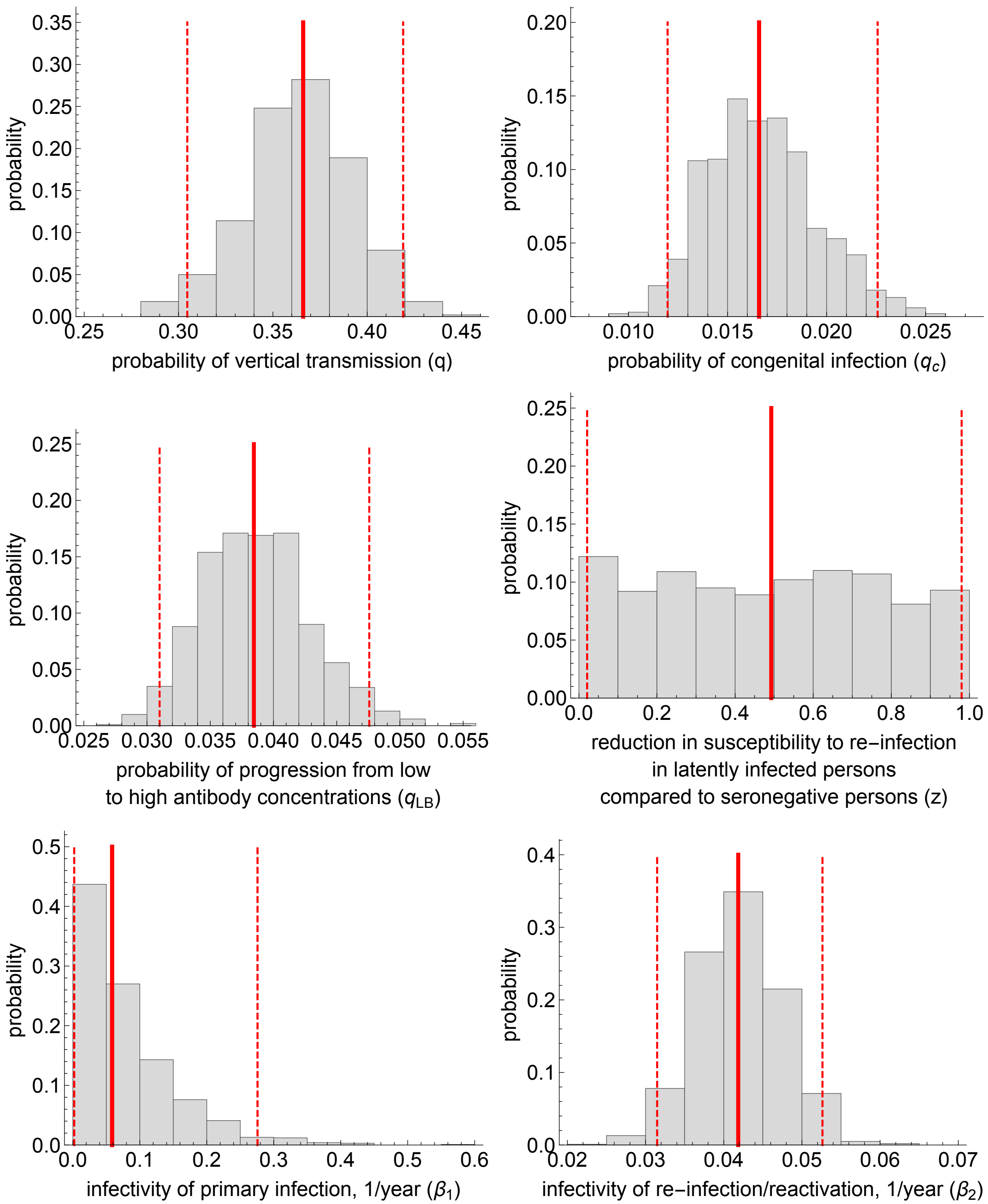

Figure S5: **Histogram of the estimated parameter values in the scenario with high reactivation rates.** The solid lines are the median estimates. The dashed lines correspond to 95% credible intervals obtained from 1000 parameter samples from the posterior distribution.

Intervention scenario	Reduction % birth prevalence cCMV median (95%CrI)	Reduction % incidence primary infection median (95%CrI)	Reduction % incidence re-infection/ reactivation median (95%CrI)	Effective reproduction number median (95%CrI)
Universal vaccination				
prevention of (re-)infection and reactivation				
6-months-old boys and girls	4.0 (3.6—4.5)	**18.7 (17.3—20.6)**	5.3 (4.3—6.0)	1.14 (1.11—1.17)
10-years-old boys and girls	10.7 (10.0—11.5)	17.5 (15.9—19.2)	7.0 (6.0—8.2)	1.15 (1.11—1.18)
10-years-old girls	10.2 (9.5—10.9)	10.7 (9.4—11.6)	4.7 (3.9—5.5)	1.19 (1.15—1.23)
25-years-old women	30.3 (30.0—30.8)	9.6 (8.6—11.0)	**7.4 (6.4—8.8)**	1.17 (1.13—1.22)
prevention of infection				
6-months-old boys and girls	2.4 (2.1—2.8)	15.3 (14.3—17.3)	2.3 (1.9—2.9)	1.18 (1.15—1.20)
10-years-old boys and girls	4.5 (3.9—5.0)	12.6 (11.6—14.0)	2.1 (1.8—2.7)	1.22 (1.19—1.25)
10-years-old girls	4.3 (3.8—4.8)	7.1 (6.4—7.8)	1.5 (1.2—1.9)	1.25 (1.22—1.28)
25-years-old women	3.9 (3.7—4.3)	4.4 (4.1—4.8)	1.0 (0.9—1.2)	1.32 (1.28—1.35)
Vaccination during pregnancy	**71.2 (71.0—71.4)**	2.7 (1.6—4.3)	5.9 (4.8—7.0)	**1.11 (1.04—1.19)**
Hygienic measures	0.9 (0.8—1.1)	2.9 (2.6—3.4)	0.7 (0.6—0.9)	1.33 (1.30—1.37)

Table S2: **Impact of interventions on cCMV, primary infection and re-infection/reactivation in the scenario with high reactivation rates.** The reductions are evaluated 20 years after the start of the intervention. The proportion of effectively vaccinated persons (vaccination coverage × vaccine efficacy) is 70%, and the average duration of protection is 10 years. Hygienic measures are modeled as a 70% reduction in infectious contacts between women of reproductive age (15-50 years) and young children (0-5 years). The effective reproduction number is defined as the average number of secondary infections at the start of an epidemic with one infected individual introduced in a population where 70% of persons are effectively vaccinated. This number smaller than 1 indicates that a given intervention is going to lead to the disease elimination in the long run.

11 DALY computation

To compute the disease burden caused by cCMV in the Netherlands, we used the yearly incidence of cCMV predicted by the model. From the yearly incidence we computed numbers of cases of cCMV per year using a population size of 17 million for the Netherlands in 2017. Furthermore, we used an estimate of the disability-adjusted life years (DALYs) per case of 3.034 (95%CrI: 1.202—6.105) from the meta-analysis for Belgium [13].The DALY per case result is not reported in the published paper, but was provided to us by Brecht Devleesschauwer (personal communication). For every vaccination scenario, we computed the yearly incidence for a period of 20 years, calculated the number of cCMV cases based on the population size of 17 million, and computed the number of DALYs per year by multiplying with the DALY per case estimate. We then calculated the number of DALYs prevented in subsequent years compared to reference year zero and added those prevented DALYs for the period of 20 years to arrive at the number of DALYs prevented over that time period.

We did the computation for the median incidence and the 95% credible interval of the incidences (which is based on the uncertainty of model parameters, but not the uncertainty in DALY per case estimate). Table S3 shows the computed numbers for one scenario, namely the vaccination of pregnant women. Figure S6 shows the DALYs prevented for all scenarios together with the 95% credible intervals based on the uncertainty in incidences.

Year	Incidence per 100,000	Number of cases in NL	Cases prevented	DALYs per 100,000	DALYs per year	DALYs prevented
0	6.84	1,163		20.75	3,527	
1	2.05	348	814	6.22	1,057	2,470.3
2	2.05	348	815	6.21	1,056	2,471.4
3	2.05	348	815	6.21	1,055	2,472.5
4	2.04	347	815	6.20	1,054	2,473.7
5	2.04	347	816	6.19	1,053	2,474.8
6	2.04	347	816	6.19	1,051	2,476.0
7	2.04	346	816	6.18	1,050	2,477.2
8	2.03	346	817	6.17	1,049	2,478.5
9	2.03	345	817	6.16	1,047	2,480.0
10	2.03	345	818	6.15	1,046	2,481.7
11	2.02	344	819	6.14	1,044	2,483.5
12	2.02	343	819	6.13	1,042	2,485.5
13	2.02	343	820	6.12	1,040	2,487.5
14	2.01	342	821	6.10	1,038	2,489.7
15	2.01	341	821	6.09	1,035	2,492.2
16	2.00	340	822	6.07	1,032	2,495.1
17	2.00	339	823	6.05	1,029	2,498.2
18	1.99	338	824	6.03	1,026	2,501.6
19	1.98	337	826	6.01	1,022	2,505.5
20	1.97	335	827	5.98	1,017	2,510.2
Totals		6,870	16,382		20,843	49,705.1

Table S3: **DALY computation for vaccination of pregnant women.**

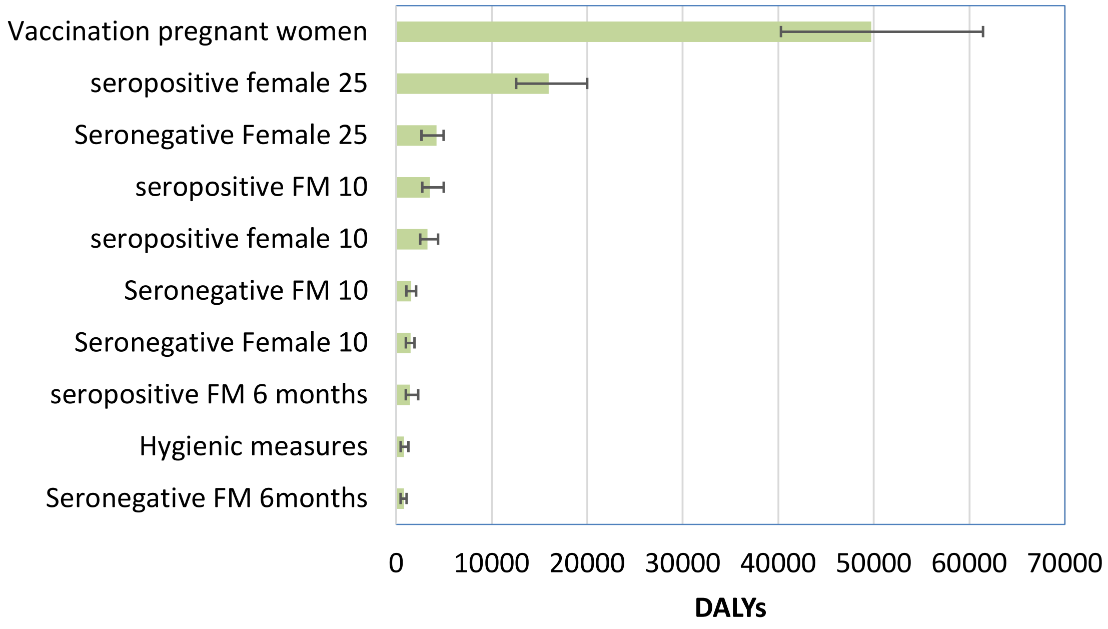

Figure S6: **DALYs prevented over 20 years for various vaccination scenarios in the Netherlands.**

×

Learn Git and GitHub without any code!

Using the Hello World guide, you'll start a branch, write comments, and open a pull request.

Read the guide

mvboven / **cmv-vaccination**

Inference part of manuscript for evaluation of vaccination against cmv

50 commits | **1** branch | **0** packages | **0** releases | **1** contributor

Branch: master | New pull request | Create new file | Upload files | Find file | Clone or download

mvboven Update README.md   Latest commit d6e6fbc on Oct 17, 2019

data	Create test	7 months ago
figures	Delete test	7 months ago
scripts	Create test	7 months ago
README.md	Update README.md	6 months ago

README.md

<table>
<tr><th>title</th><th>author</th><th>date</th><th>output</th></tr>
<tr><td>Short- and long-term impact of vaccination against cytomegalovirus: A data-driven modelling study</td><td>Michiel van Boven</td><td>4/9/2019</td><td><table><tr><th>html_document</th></tr><tr><td><table><tr><th>keep_md</th><th>highlight</th><th>number_sections</th><th>toc</th></tr><tr><td>true</td><td>haddock</td><td>true</td><td>false</td></tr></table></td></tr></table></td></tr>
</table>

Updated: 17 October 2019

Introduction

On this page I provide an overview of parameter inference using the endemic equilibrium of a transmission model tailored to the epidemiology of cytomegalovirus in high-income countries. The model includes vertical transmission (congenitally and postnatally by breastfeeding/transfer of saliva), primary infection to uninfected persons, re-infection to latently infected persons, and infectious reactivation. The analyses are based on an earlier model (https://journals.plos.org/ploscompbiol/article?id=10.1371/journal.pcbi.1005719), but with the following extensions:

- Estimation of reactivation rates is not based on piecewise constant functions anymore but on cubic splines, using code by M. Kharratzadeh (https://mc-stan.org/users/documentation/case-studies/splines_in_stan.html).
- For increased biological realism, the new transmission model allows for multiple re-infection and reactivation events during a person's lifetime.

- Data from a birth prevalence study are included, and the model is extended to be able to estimate the probability of congenital transmission. This is an important extension as much of the health burden is due to congenital infections (which can lead to serious long-term sequelae).

The analyses and results presented here form the starting point of a manuscript that evaluates the impact of various vaccination strategies against CMV. Authors of the manuscript are Ganna Rozhnova, Mirjam Kretzschmar, Fiona van der Klis, Debbie van Baarle, Marjolein Korndewal, Ann Vossen, and Michiel van Boven.

Data

All data have been described and published elsewhere, and are available in the data directory or R script (https://github.com/mvboven/cmv-vaccination).

- The serological data have been analysed earlier (see https://www.sciencedirect.com/science/article/pii/S1386653214004612, https://journals.plos.org/ploscompbiol/article?id=10.1371/journal.pcbi.1005719, and the PhD thesis of M. Korndewal, available at https://openaccess.leidenuniv.nl/handle/1887/45778). As in our earlier analyses, from the serological study we select persons of Western ethnicity (ethnicity is a known independent risk factor for infection and the majority of persons in the Netherlands are of Western ethnicity), and exclude infants under 6 months (to prevent interference of maternal antibodies). Further, a subset of 651 samples are right-censored, so 2,842 and 2,337 samples from female and male participants are included in the analyses.
- The cCMV birth prevalence data also have been described earlier (see https://www.cambridge.org/core/journals/epidemiology-and-infection/article/disease-burden-of-congenital-cytomegalovirus-infection-at-school-entry-age-study-design-participation-rate-and-birth-prevalence/DED925A2D496FF0ED8DCDCA5E692AFB2/core-reader, and in particular the PhD thesis of M. Korndewal, available at https://openaccess.leidenuniv.nl/handle/1887/45778). In the birth prevalence study, 154 infants of a cohort of 31484 (0.5%) are found positive. This is broadly in line with findings from other high-income countries.
- Human contact data (stratified by sex, 16 age groups of 5 years) are as in our previous study. See https://github.com/kassteele/Contact-patterns for explanation, alternative options, and do-it-yourself instructions.
- Ages of mothers in the Netherlands in 2006/2007 are taken from Statistics Netherlands (http://www.cbs.nl), see also the MSc thesis of Sophia de Jong (available on request).

Stan model

Data

Data and fixed parameter values are specified in the data directory (serological data and contact matrix) and R script (all other). Throughout, I take the estimated means and standard deviations of the mixing distributions as estimated in our earlier analyses. The data block of the Stan model looks as follows:

```
data {
  int<lower=0> N;                           // number of subjects
  int<lower=1> DeltaA;                      // length of age intervals
  int<lower=1> A;                           // number of age intervals
  matrix<lower=0>[A, A] Contact_MM;         // gender- and age-specific contact matrices
  matrix<lower=0>[A, A] Contact_FM;         // male to female contact matrix
  matrix<lower=0>[A, A] Contact_MF;         // female to male contact matrix
  matrix<lower=0>[A, A] Contact_FF;         // female to female
  int<lower=0, upper=DeltaA*A> Ages[N];     // subject ages (unit: years; precision: 1/12)
  real Titers[N];                           // antibody titers
  int Censor[N];                            // 0 = normal, 1 = censored, 2 = spike (see mvb17)
  real RightCensor;                         // titers above this value are right-censored
  real MuS;                                 // mean of classification mixture (S) (estimated in mvb17)
  real MuL;                                 // mean of classification mixture (L)
  real MuB;                                 // mean of classification mixture (B)
  real<lower=0> SigmaS;                     // standard deviation of the uninfected component
  real<lower=0> SigmaL;                     // standard deviation of the infected component
  real<lower=0> SigmaB;                     // standard deviation of infected with raised antibodies
  int<lower=1> numbertestedinfants;         // # infants tested for cCMV - total: 31,484
  int<lower=1> numbercCMVinfants;           // # infants positive for cCMV - total: 154
  real<lower=0> Penalty;                    // estimation of the fois/S0: LHS ~ N(RHS,1/Penalty)
  int<lower=0, upper=1> Gender[N];          // 0 = female, 1 = male
```

```
  vector[A] BirthContribution;              // prob dist of ages of mothers with a newborn in 2006/2007
  int num_knots;                            // number of spline knots
  vector[num_knots] knots;                  // the sequence of knots
  int spline_degree;                        // the spline degree (order - 1)
  real ts[DeltaA*A];                        // ages at which splines are calculated
  real<lower=0, upper=1> reducinf;          // infectivity reduction in L compared to B
  int<lower=0, upper=1> mode;               // 0 = regular sampling, 1 = sampling to compute WBIC
}
```

Parameters

Fundamental parameters of the model are the infectiousness of persons with a primary infection (beta_1), the infectiousness after re-infection or reactivation (beta_2), the reduced susceptibility to re-infection as compared with primary infection (z), the probability that re-infection or reactivation lead to antibody boosting (probLtoB), the fraction of the population that is uninfected at 6 months (i.e. not infected congenitally or postnatally) (S0), and the probabilities of congential infection from an acutely infected mother (qcCMV) and postnatal infection (e.g., by breastfeeding, transfer of saliva from mother to offspring) (nu). Also estimated are the spline wieghts for the reactivation rates in females and males (a_raw). The 16x2=32 nonlinear equations for the age-specific forces of infection at equilibrium (lambda_f and lambda_m) that result after interval decomposition (16 for each sex; see e.g., Chapter 9 of https://press.princeton.edu/titles/9916.html for details) are efficiently solved using a trick (see below). The parameter block is given by:

```
parameters {
  real<lower=0> beta1;                      // infectivity after primary infection
  real<lower=0> beta2;                      // infectivity after reactivation/re-infection in L or L/B
  real<lower=0, upper=1> z;                 // reduction in susceptibility to reinfection
  real<lower=0, upper=1> probLtoB;          // prob that reactivation/reinfection leads to Ab boosting
  real<lower=0> S0;                         // fraction of the population not vertically infected
  real<lower=0, upper=1> qcCMV;             // prob cCMV during acute infection of mother
  real<lower=0> nu;                         // prob vertical transmission (including cCMV)
  matrix<lower=0>[2, num_basis] a_raw;      // spline basis functions; 1 = female, 2 = male
  vector<lower=0>[A] lambda_f;              // forces of infection on the age-intervals in females
  vector<lower=0>[A] lambda_m;              // forces of infection on the age-intervals in males
}
```

Analysis

The transformed parameters block contains the specifications of the sex- and age-specific reactivation rates on age intervals, and the age-specific fractions in the S, L, or B compartments at points of the age intervals:

```
/* reactivation rates on age intervals */
vector<lower=0>[DeltaA*A] rho_f;            // reactivation rate in females on intervals
vector<lower=0>[DeltaA*A] rho_m;            // reactivation rate in females on intervals

/* prevalence in S, L, and B at points of age intervals */
vector<lower=0, upper=1>[DeltaA*A+1] S_f;     // susceptible prevalence at points of intervals (female)
vector<lower=0, upper=1>[DeltaA*A+1] S_m;     // susceptible prevalence at points of intervals (male)
vector<lower=0, upper=1>[DeltaA*A+1] L_f;     // latently infected (female)
vector<lower=0, upper=1>[DeltaA*A+1] L_m;     // latently infected (male)
vector<lower=0, upper=1>[DeltaA*A+1] B_f;     // infected with boosted titers (female)
vector<lower=0, upper=1>[DeltaA*A+1] B_m;     // infected with boosted titers (male)
```

In addition, the transformed parameters block contains specifications of auxiliary parameters for efficient solution of the discretised ODEs (see below), and forces of infection on the (broader) age intervals for which the contact matrix is specified:

```
/* auxiliary vectors to efficiently solve ODEs for S, L, and B */
vector<lower=0, upper=1>[DeltaA*A+1] X_f;     // boys latently infected at birth
vector<lower=0, upper=1>[DeltaA*A+1] X_m;     // girls latently infected at birth
vector<lower=0>[DeltaA*A+1] Y_f;              // =(L_f-X_f)/X_f ratio in L infected hor/vert
vector<lower=0>[DeltaA*A+1] Y_m;              // =(L_m-X_m)/X_m

/* lambda hat (i.e. the force of infection) should be very similar to lambda */
vector<lower=0>[A] lambda_hat_f;
vector<lower=0>[A] lambda_hat_m;
```

Next, the main steps are as follows:

- First, the ODEs for the age- and sex-specific prevalence at equilbrium are solved in terms of the forces of infection and other parameters. Details are available on request.
- Second, the resulting equations are discretised on a fine-grained mesh (now 1 year), assuming that rate parameters (i.e. reactivation rates) are constant on the intervals. The mesh can be made even more fine grained, or interpolations can be used to make the likelihood contributions of the serological data more precise. In our experience, there is little additional precision to be gained by such approaches. See below for code relating to the first two steps.

```
/* solution of the ODEs S, L, B, and intermediates X/Y in terms of the foi in females and males      */
/* X : perinatally infected and still in L                                                           */
/* Y : ratio of persons in L that are infected after birth (L-X) over those infected perinatally (X) */

S_f = S0 * exp(-cumulative_sum(append_row(Zero, longLambda_f)));
S_m = S0 * exp(-cumulative_sum(append_row(Zero, longLambda_m)));

X_f = (1.0 - S0) * exp(-cumulative_sum(append_row(Zero, probLtoB * longPi_f)));
X_m = (1.0 - S0) * exp(-cumulative_sum(append_row(Zero, probLtoB * longPi_m)));

Y_f = cumulative_sum(append_row(Zero, longLambda_f .* (S_f[:DeltaA*A] ./ X_f[:DeltaA*A])
  .* (LongOnes - exp(-(longLambda_f - probLtoB * longPi_f))) ./ (longLambda_f - probLtoB * longPi_f)));
Y_m = cumulative_sum(append_row(Zero, longLambda_m .* (S_m[:DeltaA*A] ./ X_m[:DeltaA*A])
  .* (LongOnes - exp(-(longLambda_m - probLtoB * longPi_m))) ./ (longLambda_m - probLtoB * longPi_m)));

L_f = X_f .* (Y_f + LlongOnes);
L_m = X_m .* (Y_m + LlongOnes);

B_f = LlongOnes - S_f - L_f;
B_m = LlongOnes - S_m - L_m;
```

- Third, the discretised solutions for the prevalence in S, L, and B are inserted in the equations for the forces of infection, while using the short-disease approximation (acute infections are 2-4 orders of magnitude shorter than human lifespan). This procedure yields 32 (16 age groups, 2 sexes) non-linear equations for the forces of infection. See below for code:

```
/* new model (compared to mvb17) that splits between infectiousness from L vs B and enables estimation
*/
/* of all parameters. It still may not entirely be biologically intuitive. A full model which distinguishes
*/
/* between infectiousness after reactivation and re-infection and allows cycling in L and B would need at
*/
/* least five infected classes.
*/
for (a in 1 : A) {
  aggr_S_f[a] = sum(longLambda_f[1+DeltaA*(a-1):DeltaA*a] .* S_f[1+DeltaA*(a-1):DeltaA*a]);
  aggr_L_f[a] = sum((rho_f[1+DeltaA*(a-1):DeltaA*a] + z * longLambda_f[1+DeltaA*(a-1):DeltaA*a])
                  .* L_f[1+DeltaA*(a-1):DeltaA*a]);
  aggr_L_m[a] = sum((rho_m[1+DeltaA*(a-1):DeltaA*a] + z * longLambda_m[1+DeltaA*(a-1):DeltaA*a])
                  .* L_m[1+DeltaA*(a-1):DeltaA*a]);
  aggr_B_f[a] = sum((rho_f[1+DeltaA*(a-1):DeltaA*a] + z * longLambda_f[1+DeltaA*(a-1):DeltaA*a])
                  .* B_f[1+DeltaA*(a-1):DeltaA*a]);
  aggr_B_m[a] = sum((rho_m[1+DeltaA*(a-1):DeltaA*a] + z * longLambda_m[1+DeltaA*(a-1):DeltaA*a])
                  .* B_m[1+DeltaA*(a-1):DeltaA*a]);
}
lambda_hat_f = Contact_FF * (beta1 * (S_f[ReduceIdxs] - S_f[ReduceIdxsRightShift]) + reducinf * beta2 *
aggr_L_f + beta2 * aggr_B_f)
             + Contact_FM * (beta1 * (S_m[ReduceIdxs] - S_m[ReduceIdxsRightShift]) + reducinf * beta2 *
aggr_L_m + beta2 * aggr_B_m);
lambda_hat_m = Contact_MM * (beta1 * (S_m[ReduceIdxs] - S_m[ReduceIdxsRightShift]) + reducinf * beta2 *
aggr_L_m + beta2 * aggr_B_m)
             + Contact_MF * (beta1 * (S_f[ReduceIdxs] - S_f[ReduceIdxsRightShift]) + reducinf * beta2 *
aggr_L_f + beta2 * aggr_B_f);
```

- Finally, the equations for the forces of infection are solved, and the result is inserted in the solution of the ODEs. Here, the above equations are solved (quite efficiently) using Stan. More precisely, taking our parameters of interest (lambda_f and lambda_m) to be random variates, calculate the right-hand sides of the equations for the forces of infection (lambda_hat_f and lambda_hat_m), and obtain approximate solutions by assuming that lambda_f and lambda_m are normally distributed with means lambda_hat_f and lambda_hat_m and very small standard deviations (1/Penalty). The code in the parameters block is as follows:

```
/* penalise the difference between lambda and lambda_hat/S0 and S0_hat to solve the equations          */
/* lambda_f - lambda_hat_f ~ normal(0,1/Penalty) does not work: do  target += log(det(Jacobian))        */
```

```
/* future: use solver. I have tried this but now still seems prohibitively slow               */
/* the formulation below can be viewed as a model in itself: A priori we would like to select parameter */
/* values that are compatible with a transmission model. Taking a high penalty, the result will match   */
/* the transmission model. For low penalty the result may differ from the transmission model.           */
/* Here, I have taken the penalty to be 10^4, which forces lambda and lambda_hat to be quite similar.   */

lambda_f ~ normal(lambda_hat_f, 1/Penalty);
lambda_m ~ normal(lambda_hat_m, 1/Penalty);
```

Priors and likelihood

Apart from the forces of infection and fraction of the population that is susceptible explicit prior distributions are provided for the spline weights. Based on earlier analyses and the expectation that reactivation is a rare event, I take Gamma(2,50) prior distributions for all weights, yielding prior expectations for the reactivation rates of 0.04 per year. In a sensitivity analysis I have included a scenario in which reactivation is expected to be (much) more frequent (taking Gamma (10,20) prior distributions), yielding prior expectations of the reactivation rates of 0.5 per year.

```
model {
  /* spline weights for the reactivation rates */
  for (s in 1:num_basis) {
   a_raw[,s] ~  gamma(2, 50);       // default, based on mvb17 and the premise that reactivation is rare
   //a_raw[,s] ~  gamma(10, 20);    // alternative, assuming that reactivation may occur frequently
  }
```

Next, the likelihood contains contributions from the serological study and birth cohort. See our earlier anaylses and the code below:

```
/* data set 1: serological data from PIENTER2 study */
for ( i in 1 : N ) {// loop over subjects
   int aa;
   real pS; real pL; real pB;

   // improve readability
 aa = Ages[i] + 1; // the index for S, L and B

   // compute the compartment-probabilities given the subjects' age
   if ( Gender[i] == 0 ) { // 0 = female
      pS = S_f[aa];
      pL = L_f[aa];
      pB = B_f[aa];
   }
   else { // 1 = male
      pS = S_m[aa];
      pL = L_m[aa];
      pB = B_m[aa];
   }

   // likelihood contributions
   if ( Censor[i] == 0 ) { // normal data
     target += watanabe_beta * log( pS * exp(normal_lpdf(Titers[i] | MuS, SigmaS)) +
                                    pL * exp(normal_lpdf(Titers[i] | MuL, SigmaL)) +
                                    pB * exp(normal_lpdf(Titers[i] | MuB, SigmaB)) ); //or log_exp_sum
   }
   else if ( Censor[i] == 1 ) { // right censored
          target += watanabe_beta * log( pS * exp(normal_lccdf(RightCensor | MuS, SigmaS)) +
                                         pL * exp(normal_lccdf(RightCensor | MuL, SigmaL)) +
                                         pB * exp(normal_lccdf(RightCensor | MuB, SigmaB)) );
        }
        else if ( Censor[i] == 2 ) { // spike
               target += watanabe_beta * log(pS);
             }
}
/* data set 2: cCMV cases from the CROCUS study (PhD thesis M Korndewal) */
target += watanabe_beta * binomial_lpmf(numbercCMVinfants | numbertestedinfants, pcCMV);
}
```

Results

Results as presented in Rozhnova et al (2019) are obtained with the following Stan settings:

```
# estimate parameters with Stan
# main analysis with gamma(2,50) priors for spline weights
fit = stan(file = 'cmv_22082019.stan',
           data = data_values,
           init = parameter_inits,
           iter = 2000,
           warmup = 1000,
           thin = 20,
           chains = 20,
           control = list(adapt_delta = 0.97, max_treedepth = 15)
)
```

Running this may take a couple of hours, so let's load an earlier result (.rda file):

```
# Load the rda object
load(file = "cmv_22082019.rda")
```

As a first check, I plot the traceplots of the main parameters:

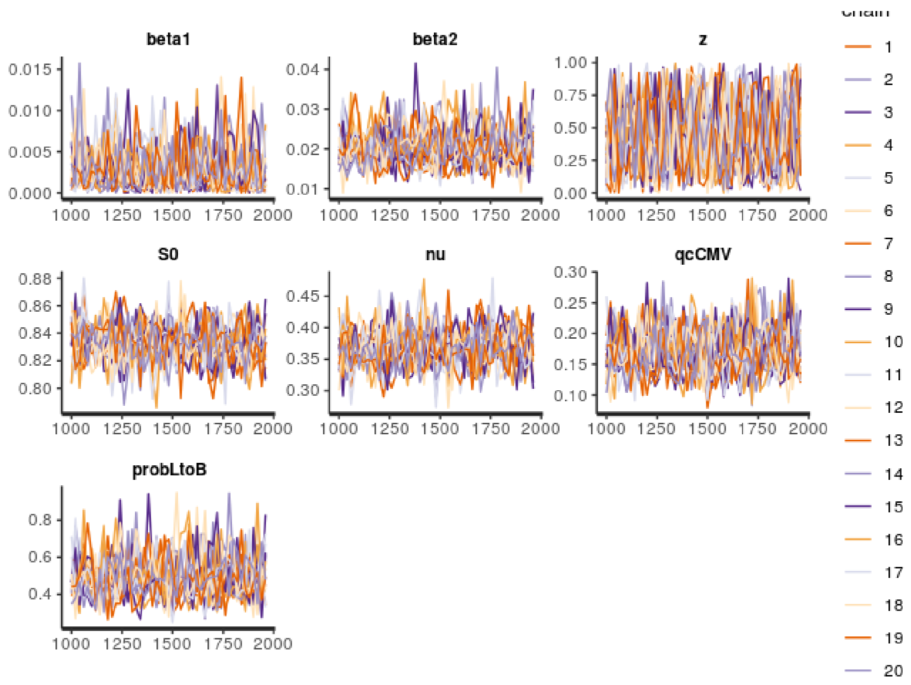

The traceplots look reasonable, so I proceed.

Posterior quantiles of the main parameters also seem reasonable (compared to earlier analyses), effective sample sizes of all parameters are around 1,000, and Rhat is close to 1 for all parameters. This is reassuring:

```
Inference for Stan model: cmv (18042019).
20 chains, each with iter=2000; warmup=1000; thin=20;
post-warmup draws per chain=50, total post-warmup draws=1000.

          mean se_mean    sd  2.5%   25%   50%   75% 97.5% n_eff  Rhat
beta1    0.003    0.000 0.003 0.000 0.001 0.002 0.004 0.010  1085 1.000
beta2    0.020    0.000 0.005 0.013 0.017 0.020 0.023 0.032  1036 1.000
z        0.446    0.008 0.278 0.029 0.203 0.420 0.666 0.953  1085 0.995
S0       0.833    0.000 0.014 0.805 0.824 0.833 0.842 0.861  1065 0.998
nu       0.369    0.001 0.030 0.311 0.349 0.370 0.389 0.429  1094 0.999
qcCMV    0.172    0.001 0.038 0.106 0.143 0.168 0.194 0.254  1082 1.001
probLtoB 0.495    0.003 0.114 0.315 0.418 0.476 0.558 0.760  1120 1.000

Samples were drawn using NUTS(diag_e) at Thu Aug 22 17:26:08 2019.
For each parameter, n_eff is a crude measure of effective sample size,
and Rhat is the potential scale reduction factor on split chains (at
convergence, Rhat=1).
```

More checks can be done by inspection of derived parameters, i.e. the forces of infection and the reactivation rates in selected age groups. Again, all seems well:

```
Inference for Stan model: cmv (18042019).
20 chains, each with iter=2000; warmup=1000; thin=20;
post-warmup draws per chain=50, total post-warmup draws=1000.

              mean se_mean    sd  2.5%   25%   50%   75% 97.5% n_eff  Rhat
lambda_f[1]  0.014   0.000 0.001 0.012 0.013 0.014 0.014 0.015  1125 1.005
lambda_f[2]  0.016   0.000 0.001 0.014 0.016 0.016 0.017 0.018  1171 1.005
lambda_f[3]  0.016   0.000 0.001 0.014 0.015 0.016 0.017 0.019  1153 0.997
lambda_f[4]  0.014   0.000 0.001 0.012 0.013 0.014 0.014 0.016  1025 0.997
lambda_f[5]  0.010   0.000 0.001 0.009 0.009 0.010 0.010 0.011   976 1.001
lambda_f[6]  0.010   0.000 0.001 0.009 0.010 0.010 0.011 0.012  1050 1.005
lambda_f[7]  0.012   0.000 0.001 0.011 0.011 0.012 0.012 0.013  1114 1.007
lambda_f[8]  0.012   0.000 0.001 0.011 0.012 0.012 0.012 0.013  1139 1.007
lambda_f[9]  0.012   0.000 0.001 0.010 0.011 0.011 0.012 0.013  1068 1.011
lambda_f[10] 0.011   0.000 0.001 0.010 0.011 0.011 0.012 0.013  1021 1.004
lambda_f[11] 0.011   0.000 0.001 0.009 0.010 0.011 0.011 0.012  1064 0.999
lambda_f[12] 0.010   0.000 0.001 0.008 0.009 0.009 0.010 0.011  1141 0.999
lambda_f[13] 0.009   0.000 0.001 0.008 0.008 0.009 0.010 0.011  1119 1.002
lambda_f[14] 0.010   0.000 0.001 0.008 0.009 0.010 0.011 0.012  1099 1.000
lambda_f[15] 0.009   0.000 0.001 0.007 0.008 0.008 0.009 0.011  1098 1.002
lambda_f[16] 0.006   0.000 0.001 0.005 0.006 0.006 0.007 0.008  1100 1.000
lambda_m[1]  0.012   0.000 0.001 0.011 0.012 0.012 0.012 0.013  1188 1.005
lambda_m[2]  0.015   0.000 0.001 0.013 0.014 0.015 0.015 0.017  1218 1.006
lambda_m[3]  0.013   0.000 0.001 0.012 0.013 0.013 0.014 0.015  1131 1.000
lambda_m[4]  0.011   0.000 0.001 0.009 0.010 0.011 0.011 0.012  1030 0.997
lambda_m[5]  0.008   0.000 0.000 0.007 0.007 0.008 0.008 0.009   909 1.001
lambda_m[6]  0.008   0.000 0.001 0.007 0.008 0.008 0.009 0.009   929 1.002
lambda_m[7]  0.010   0.000 0.001 0.009 0.010 0.010 0.010 0.011  1033 1.008
lambda_m[8]  0.012   0.000 0.001 0.011 0.012 0.012 0.013 0.014  1055 1.007
lambda_m[9]  0.011   0.000 0.001 0.010 0.011 0.011 0.012 0.013  1035 1.012
lambda_m[10] 0.010   0.000 0.001 0.009 0.010 0.010 0.011 0.012  1003 1.005
lambda_m[11] 0.010   0.000 0.001 0.008 0.009 0.010 0.010 0.011  1007 1.002
lambda_m[12] 0.009   0.000 0.001 0.008 0.009 0.009 0.010 0.011  1056 1.000
lambda_m[13] 0.011   0.000 0.001 0.009 0.010 0.011 0.011 0.013  1074 0.997
lambda_m[14] 0.009   0.000 0.001 0.007 0.009 0.009 0.010 0.012  1131 0.999
lambda_m[15] 0.008   0.000 0.001 0.006 0.008 0.008 0.009 0.011  1127 1.003
lambda_m[16] 0.008   0.000 0.001 0.006 0.007 0.008 0.009 0.011  1155 1.005
rho_f[25]    0.042   0.000 0.012 0.024 0.034 0.041 0.049 0.068   995 1.003
rho_f[50]    0.059   0.000 0.016 0.033 0.048 0.057 0.069 0.095  1038 1.005
rho_f[75]    0.049   0.001 0.019 0.021 0.035 0.047 0.060 0.092  1130 1.001
rho_m[25]    0.023   0.000 0.008 0.011 0.018 0.022 0.028 0.042  1042 0.997
rho_m[50]    0.025   0.000 0.008 0.011 0.019 0.024 0.029 0.043  1077 0.999
rho_m[75]    0.038   0.000 0.015 0.013 0.027 0.036 0.048 0.072  1131 0.998

Samples were drawn using NUTS(diag_e) at Thu Aug 22 17:26:08 2019.
For each parameter, n_eff is a crude measure of effective sample size,
and Rhat is the potential scale reduction factor on split chains (at
convergence, Rhat=1).
```

Pair plots of the main parameters uncover strong correlations between some parameters, and substantial differences in precision of estimates. For instance, the reduction in susceptibiliy to re-infection as compared to primary infection (z) cannot be estimated with any meaningful precision, while there is a strong correlation between the fraction of of the population that is stil susceptible at 6 months (S0), and the probability of vertical transmission (nu):

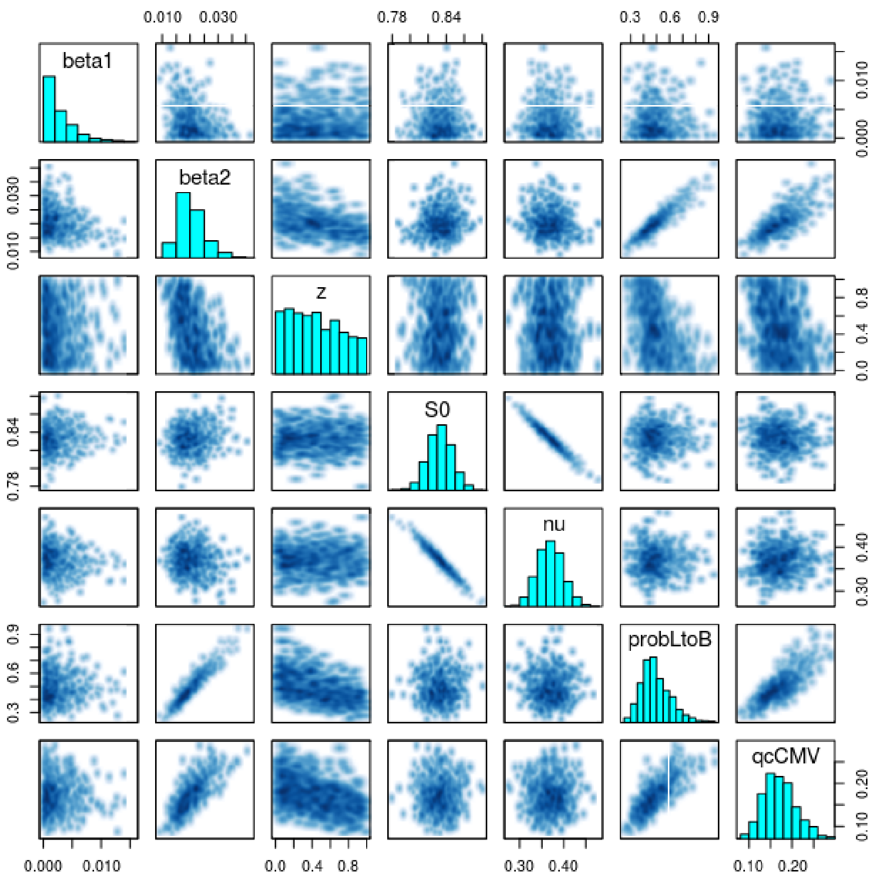

The above results make intuitive sense, and can be explained in biological terms.

It is convenient to have the maximum a posteriori (MAP) estimate at hand for forward simulations from the endemic equilibrium. The MAP is also used to draw some of the figures in the manuscript:

```
# extract parameters
params = rstan::extract(fit)
# max(params$lp__)
posmax <- as.numeric(which(params$lp__ == max(params$lp__)))
MAP <- as.data.frame(fit)[posmax, c("beta1",
                                    "beta2",
                                    "z",
                                    "S0",
                                    "nu",
                                    "probLtoB",
                                    "qcCMV")]
print(MAP, digits = 4)
```

```
      beta1    beta2      z     S0     nu probLtoB  qcCMV
721 0.00677 0.01792 0.3779 0.8583 0.321    0.491 0.1384
```

For model selection based on (approximations of) leave-one-out predictive performance, I use the loo package (see, e.g., https://mc-stan.org/loo/ and references on that page):

```
LL = extract_log_lik(fit, parameter_name = 'log_lik')
loo(LL)
```

```
Warning: Relative effective sample sizes ('r_eff' argument) not specified.
For models fit with MCMC, the reported PSIS effective sample sizes and
MCSE estimates will be over-optimistic.
```

```
Computed from 1000 by 5179 log-likelihood matrix
```

```
         Estimate    SE
elpd_loo -11071.8 184.4
p_loo         7.6   0.2
looic     22143.6 368.8
------
Monte Carlo SE of elpd_loo is 0.1.

All Pareto k estimates are good (k < 0.5).
See help('pareto-k-diagnostic') for details.
```

Finally, let's visualise the estimated prevalence in females and males, as well as the reactivation rates. The estimated prevalence are in good agreement with our earlier estimates (as they should). As in our earlier study, reactivation rate estimates are generally higher in females than in males.

Female prevalence:

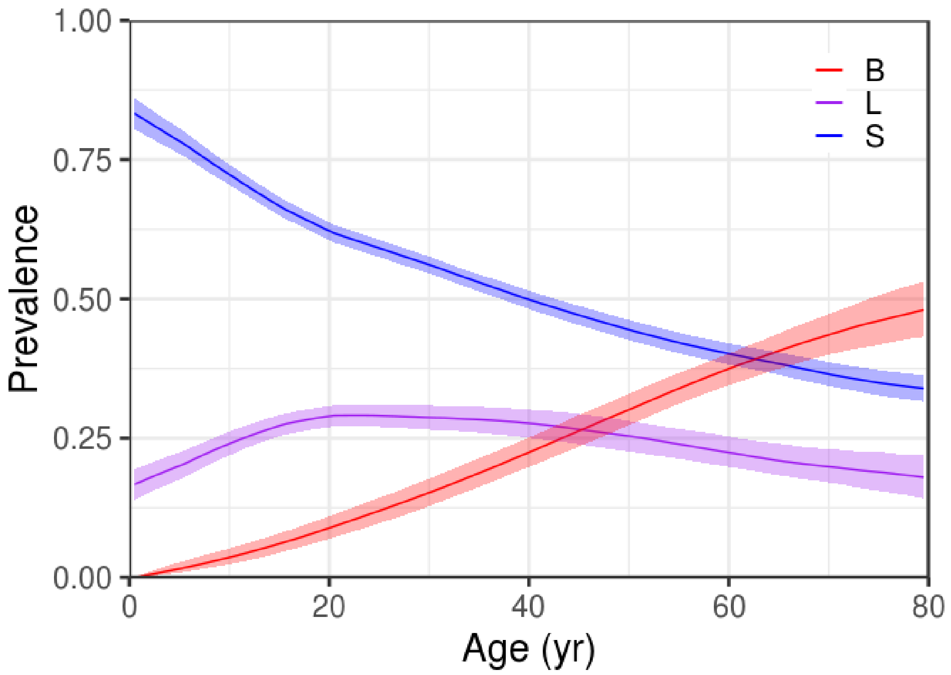

Male prevalence:

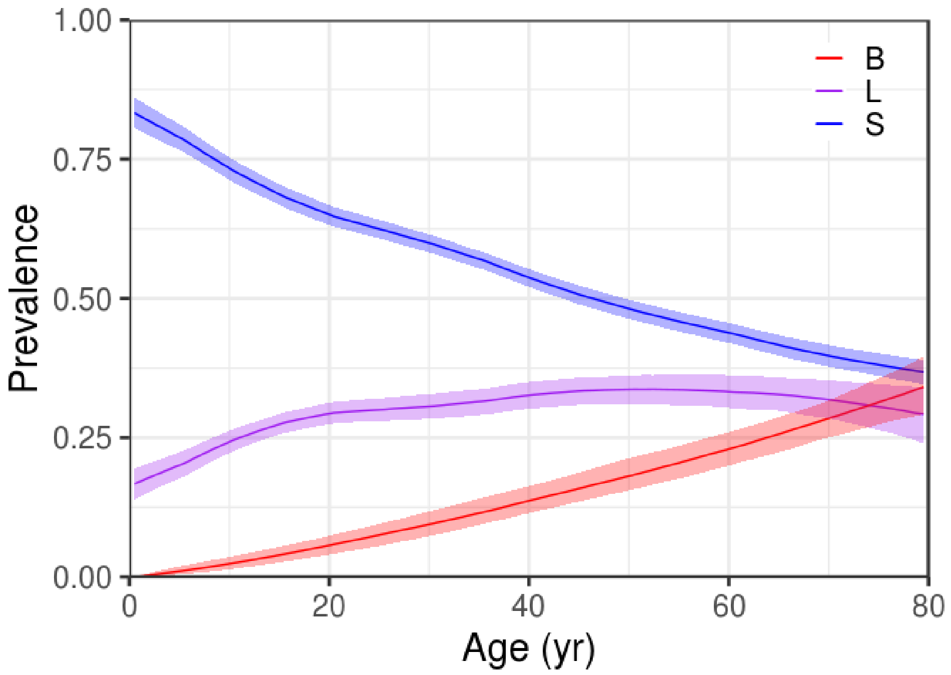

Reactivation rates:

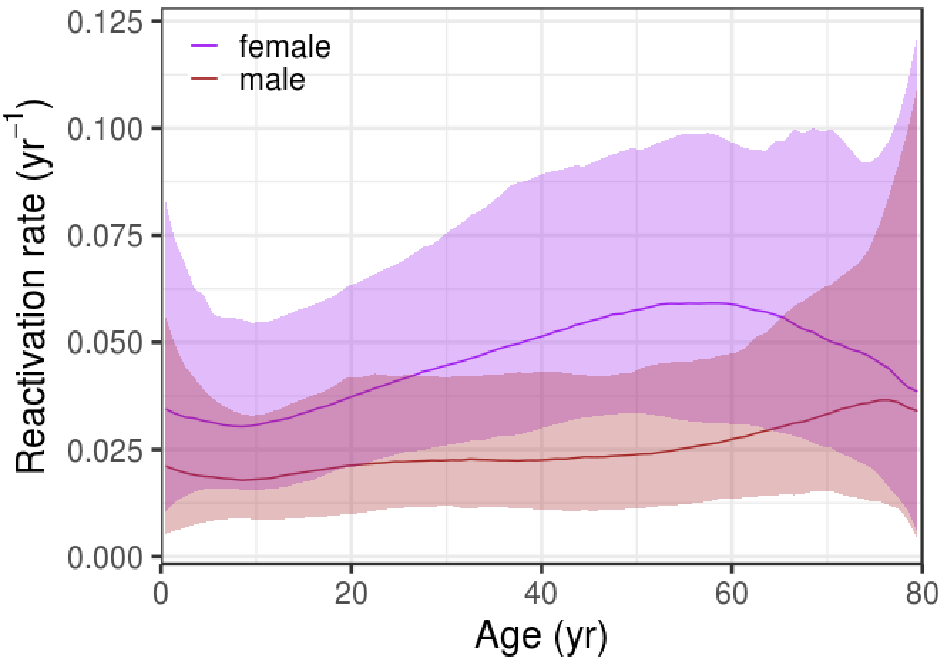

Sensitivity analysis

Speaking from experience, the results (i.e. parameter estimates) are remarkably robust to variations in the prior distributions. In fact, explicit prior distributions are only specified for spline weights. The prior distributions for the weights do have a strong impact on the parameter estimates. Therefore, I show results for an alternative scenario with higher (gamma(10,20)) prior distributions for the splines weights. Results form the default scenario are removed, and an alternative scenario is loaded:

```
# Load the rda object
rm(fit)
rm(params)
load(file = "high reactivation_10052019.rda")
params = rstan::extract(fit)
```

Again, the traces, effective sample sizes, Rhat, and pairplots suggest that the fitting procedure has delivered good results:

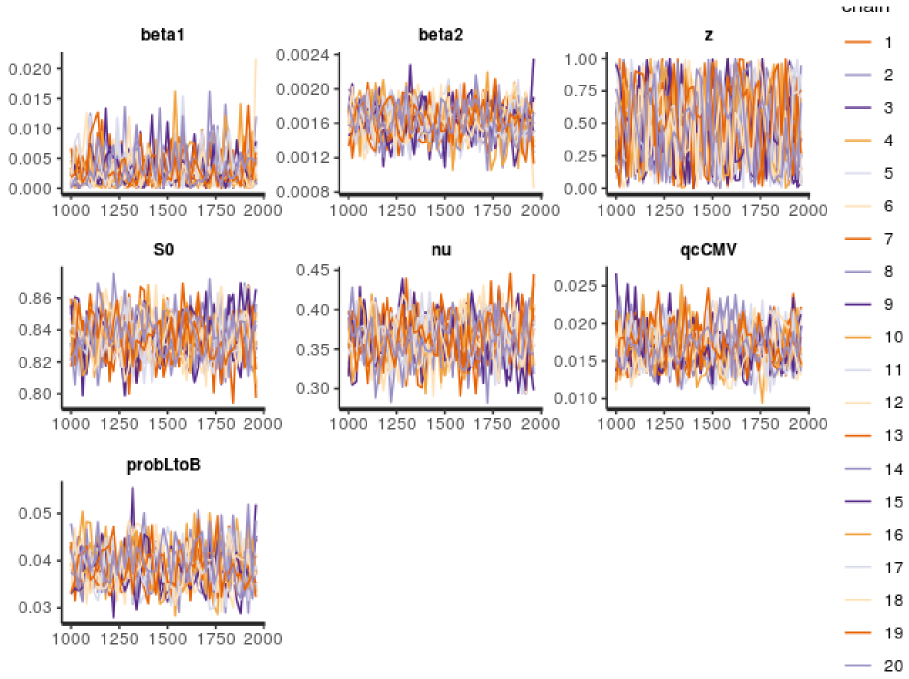


```
Inference for Stan model: cmv (18042019).
20 chains, each with iter=2000; warmup=1000; thin=20;
post-warmup draws per chain=50, total post-warmup draws=1000.
```

```
           mean se_mean    sd  2.5%   25%   50%   75% 97.5% n_eff  Rhat
beta1     0.003   0.000 0.003 0.000 0.001 0.002 0.004 0.011  1159 1.007
beta2     0.002   0.000 0.000 0.001 0.001 0.002 0.002 0.002  1138 1.000
z         0.485   0.009 0.291 0.023 0.223 0.493 0.728 0.980  1036 0.994
S0        0.835   0.000 0.013 0.810 0.826 0.835 0.844 0.863  1082 0.996
nu        0.364   0.001 0.028 0.305 0.346 0.366 0.382 0.419  1032 0.998
qcCMV     0.017   0.000 0.003 0.012 0.015 0.017 0.018 0.023  1016 1.004
probLtoB 0.039   0.000 0.004 0.031 0.036 0.038 0.041 0.048  1056 0.999

Samples were drawn using NUTS(diag_e) at Thu May  9 18:39:18 2019.
For each parameter, n_eff is a crude measure of effective sample size,
and Rhat is the potential scale reduction factor on split chains (at
convergence, Rhat=1).
```

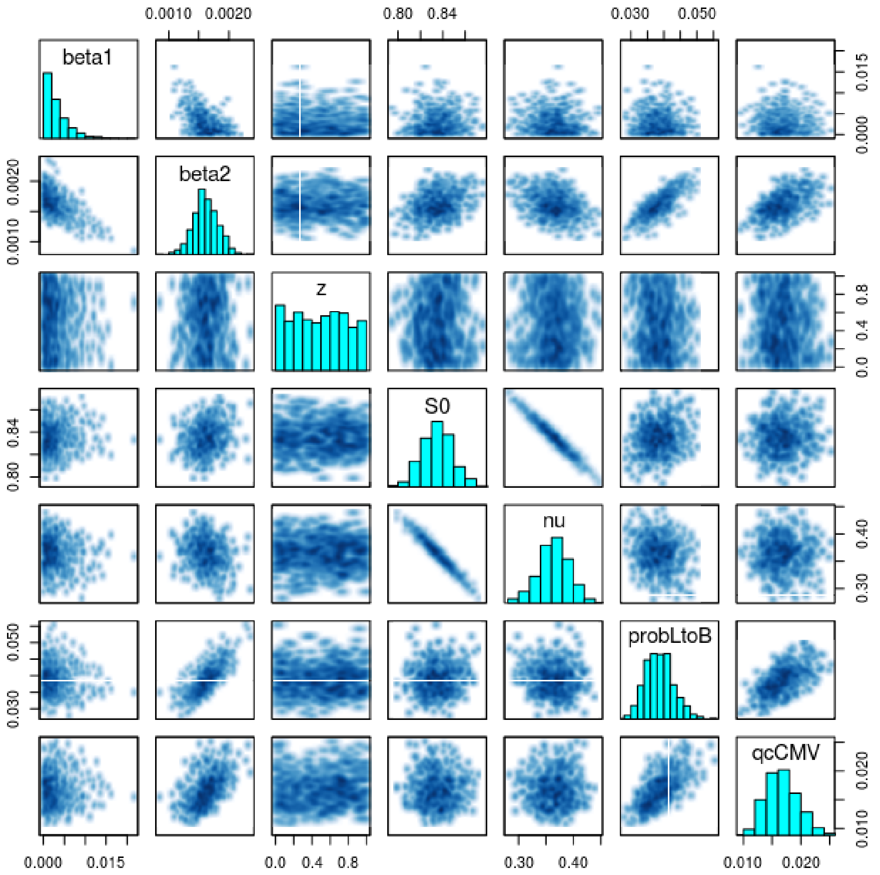

LOOIC suggests that the variant model has slightly higher statistical support:

```
LL = extract_log_lik(fit, parameter_name = 'log_lik')
loo(LL)
```

```
Warning: Relative effective sample sizes ('r_eff' argument) not specified.
For models fit with MCMC, the reported PSIS effective sample sizes and
MCSE estimates will be over-optimistic.
```

```
Computed from 1000 by 5179 log-likelihood matrix

         Estimate    SE
elpd_loo -11071.3 184.4
p_loo         5.5   0.1
looic     22142.7 368.8
------
Monte Carlo SE of elpd_loo is 0.1.

All Pareto k estimates are good (k < 0.5).
See help('pareto-k-diagnostic') for details.
```

Next, as in the default scenario let's visualise the estimated prevalences in females and males, as well as the reactivation rates.

Female prevalence:

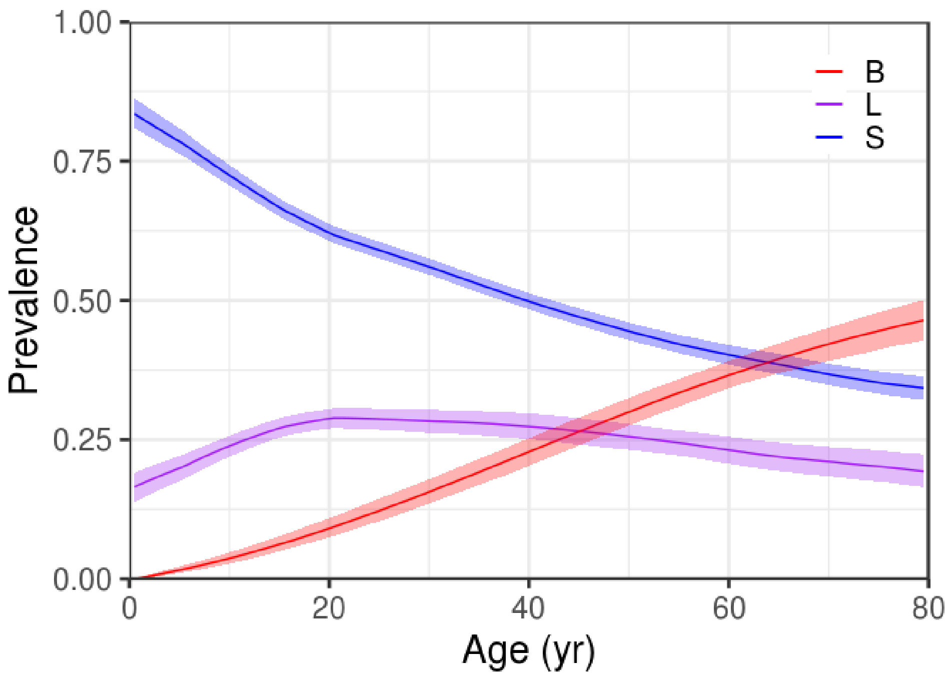

Male prevalence:

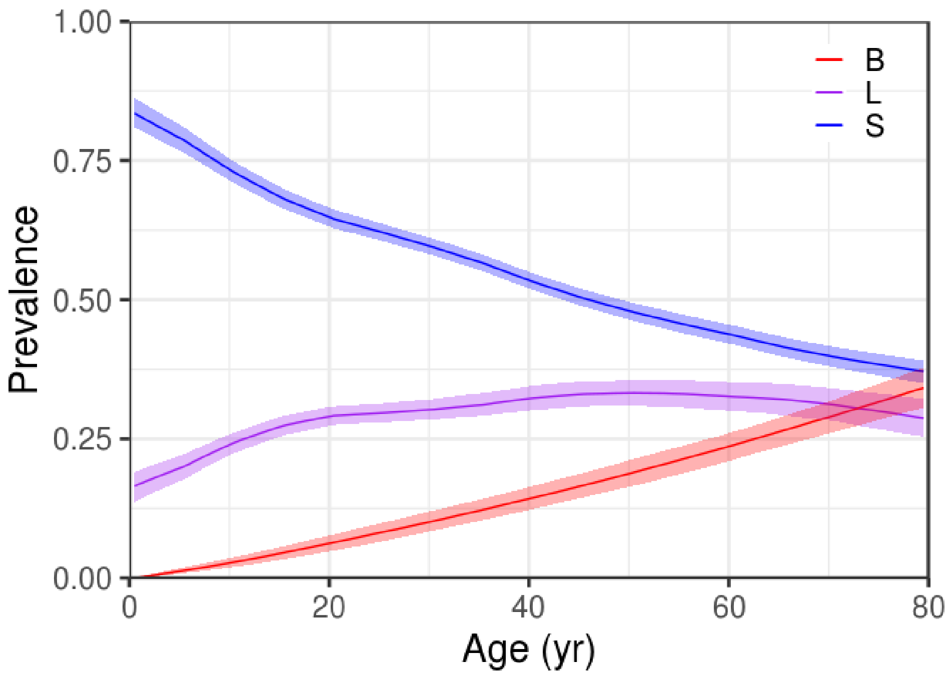

Reactivation rates:

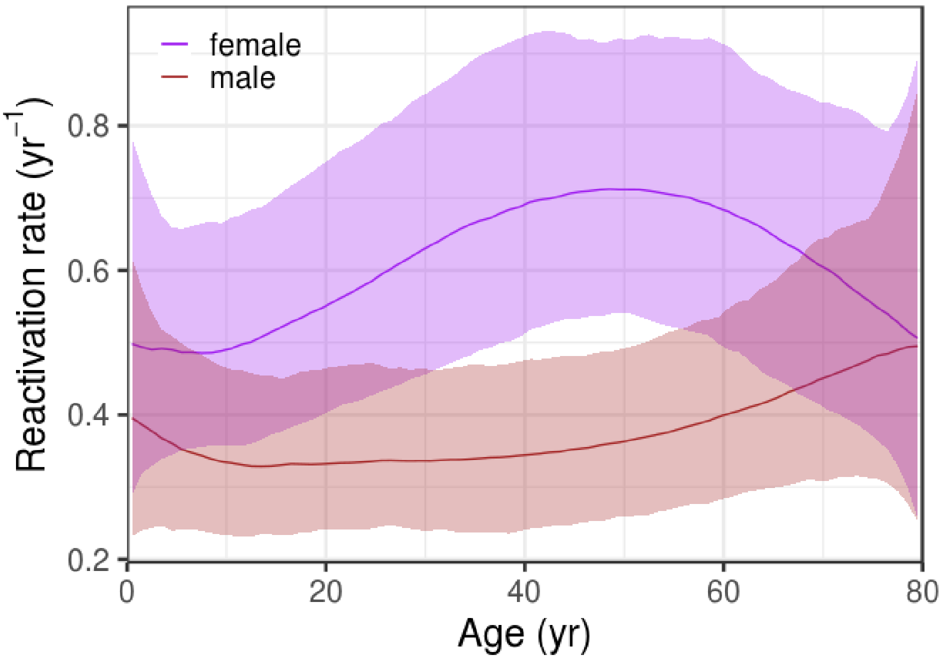

Notice that there is very good correspondence for the prevalence in both sexes between the two scenarios, but that the reactivation rate is much higher in the scenario with high spline priors. The increase in the reactivation rates is offset by a concomitant decrease in the transmissibilities of primary infection and re-infection/reactivation (beta1 and beta2).
